# The State-of-the-Art Mechanisms and Antitumor Effects of Somatostatin in Colorectal Cancer: A Review

**DOI:** 10.3390/biomedicines12030578

**Published:** 2024-03-05

**Authors:** Aldona Kasprzak, Agnieszka Geltz

**Affiliations:** Department of Histology and Embryology, University of Medical Sciences, Swiecicki Street 6, 60-781 Poznań, Poland; szumigala.agnieszka@gmail.com

**Keywords:** neuroendocrine and non-endocrine tumors, colorectal cancer (CRC), somatostatin (SST, SRIF), somatostatin analogues (SSAs), mechanisms of antitumor activity, SST/SSAs in cancer therapy

## Abstract

Somatostatin, a somatotropin release inhibiting factor (SST, SRIF), is a widely distributed multifunctional cyclic peptide and acts through a transmembrane G protein-coupled receptor (SST1-SST5). Over the past decades, research has begun to reveal the molecular mechanisms underlying the anticancer activity of this hormonal peptide. Among gastrointestinal tract (GIT) tumors, direct and indirect antitumor effects of SST have been documented best in gastroenteropancreatic neuroendocrine tumors (GEP-NETs) and less well in non-endocrine cancers, including sporadic colorectal cancer (CRC). In the latter, the signaling pathways involved in the antitumor function of SST are primarily MAPK/ERK/AKT and Wnt/β–catenin. Direct (involving the MAPK pathway) and indirect (VEGF production) antiangiogenic effects of SST in CRC have also been described. The anti-inflammatory role of SST in CRC is emphasized, but detailed molecular mechanisms are still being explored. The role of SST in tumor genome/tumor microenvironment (TME)/host’s gut microbiome interactions is only partially known. The results of SST analogues (SSAs)’ treatment of sporadic CRC in monotherapy in vivo are not spectacular. The current review aims to present the state-of-the-art mechanisms and antitumor activity of endogenous SST and its synthetic analogues in CRC, with particular emphasis on sporadic CRC.

## 1. Introduction

Colorectal cancer (CRC), which encompasses the colorectum (including the anus), referred to as ICD-10 C18-C21 [1], is the third most prevalent malignant tumor around the world (including Europe) and second among cancer-related death causes [2,3,4]. In Poland in 2020, in terms of incidence, it also ranked third among both sexes. As a cause of mortality, it was in second place in men and third in women [3]. About 90% of CRC cases are sporadic, caused by somatic mutations leading to invasive cancer [5].

Antitumor effects include primarily inhibiting cell proliferation and increasing cell apoptosis. Other processes (often associated with antiproliferative effects) include inhibition of angiogenesis and regulation of the tumor’s immune status [6,7]. In the case of CRC, potential antitumor effects also include eliminating chronic inflammatory changes, modifying the intestinal microbiome and regulating the intestinal barrier and the interaction between cancer and the tumor microenvironment (TME) [8,9,10,11,12]. Somatostatin, a somatotropin release inhibiting factor (SST, SRIF), discovered in the rat/ovine hypothalamus [13,14], is a widely distributed multifunctional cyclic peptide produced by cells scattered throughout the human body [15,16]. SST as an endogenous peptide hormone and its synthetic analogues (SSAs) acts through five types of SST receptors (SSTRs): SST1, SST2 (SST2A and SST2B in rodents) [17,18], SST3, SST4 and SST5, which belong to the superfamily of transmembrane G protein-coupled receptors (GPCR family). Functionally, SST and SSAs are related to well-known signal transduction pathways presented in other reviews [15,19,20,21,22,23].

The most commonly described effects of somatostatin receptor (SST1-5) activation by ligand (SST) are: (i) adenylyl cyclase (ACL) inhibition; (ii) activation of protein phosphotyrosine phosphatases (PTPs); and (iii) modulation of mitogen-activated protein kinase/extracellular signal regulated kinase (MAPK/ERK). Other antitumor effectors via SSTRs include (iv) phosphatidylinositol 3 kinase (PI3K)/protein kinase B (AKT) and (v) calcium signaling pathways [21,22,23]. ACL inhibition leads to a decrease in adenosine monophosphate (cAMP), which results in the downregulation of protein kinase activity, which suppresses the activity of oncogenes and the development of cancer. Activation of PTPs leads to dephosphorylation and inactivation of tyrosine kinase [24]. Among the protein kinases inhibited by PTPs is MAPK, which results in inhibition of DNA and protein synthesis. This signaling pathway is also responsible for pro-apoptotic effects. In the PI3K/AKT pathway, there is an increase in the expression of p21, p27 and the tumor suppressor gene *Zac1*, which causes the accumulation of cells in the G1 phase without entering the S phase and the inhibition of cell proliferation. ACL inhibition is responsible for the antisecretory effect of SST/SSAs, as well as lowering the intracellular calcium concentration due to the inhibition of voltage-dependent Ca^2+^ channels and activation of K^+^ channels. These activities may also lead indirectly to the inhibition of proliferation [15,16,21,22,25]. The molecular mechanisms of the antitumor effects of SST have been described in various solid tumors [23,26,27,28,29].

Concerning gastrointestinal tract (GIT) tumors, the antitumor activity of SST has been well documented in gastroenteropancreatic neuroendocrine tumors (GEP-NETs) [23,30,31] and poorly in non-endocrine cancers, including sporadic CRC. There are many unresolved issues in research on the biological effects of SST in CRC. It is unclear, among others, the role of SST in CRC cell histogenesis [32,33,34,35,36] and in increasing the population of cancer stem cells (CSCs) [37,38]. Although SST was among the nine hub genes associated with the diagnosis and prognosis of CRC [39], its diagnostic and prognostic role in CRC in everyday clinical practice is poorly defined. The clinical value of examining circadian serum SST levels in CRC is also uncertain [40,41].

SSAs treatment mainly concerns highly differentiated neuroendocrine neoplasms (NENs) of GIT and other tumors that express SSTRs. Although the importance of SSAs in the treatment of symptomatic hormonally active tumors is widely recognized, their role as anticancer drugs is controversial and still undefined [42,43,44,45,46].

The current review aims to present the state-of-the-art mechanisms and antitumor activity of endogenous SST and its synthetic analogues in CRC, with particular emphasis on sporadic CRC.

## 2. Cellular Sources and Function of Somatostatin in Normal Colon

Modern research confirms previous discoveries regarding the location of SST in the large intestine [47]. They consider this hormonal peptide and neuropeptide (NP) as one of the four most common hormones detected in the mucosa of the human colon and rectum in epithelial endocrine cells/enteroendocrine cells (EECs) using the immunohistochemical (IHC) method [38,48,49,50]. Among the EECs, the cells were initially immunoreactive to 5-hydroxytryptamine (5-HT, serotonin), as well as the glucagon and bovine pancreatic peptide (BPP). Ultrastructural studies identified four main types (EC1, L, F and H) and three rare types of EECs (D, N and P). D cells corresponded to SST-producing cells [47]. According to the current nomenclature, D cells constitute the seventh cluster of a small population of EECs (3–5%) in the lower GIT in humans and various animal species [50]. The greatest number of SST-positive cells are detected in the bottom half crypt region. However, the proportion of these cells to the total number of crypt epithelial cells is very low, lower than chromogranin-positive cells [38].

What is important to emphasize is that intestinal SST, unlike gastric SST, is mostly produced in the structures of the enteric nervous system (ENS) of the submucosal and myenteric plexuses [51,52,53]. In the human colon, as in the small intestine, there is colocalization of SST and calretinin. This points to type II neurons as the primary source of SST [53]. SST expression was also detected in the rectal mucosa, although in a lower proportion of endocrine cells compared to BPP-, human PP- and glucagon-like immunoreactive cells [54].

Only a few studies concern the role of SST in the functioning of the normal human large intestine. SST-immunoreactive fibers on submucosal, but not mesenteric vessels, were observed in a healthy human colon, which could suggest the role of this peptide in the control of blood flow to the human gut [55]. In an animal model (*SST2* knockout mice), it was shown that the motor and sensory effects of SST in the colon are likely to be mediated by SST1 and SST2, with SST1 located in the longitudinal colonic smooth muscle and SST2 in the circular [56]. The inhibitory effect of SST on colonic contraction via SST5 is mentioned [19,57]. The opposite effect (enhancement of large intestinal motility) was also demonstrated in rats, which could explain the occurrence of chronic abdominal pain and impaired motility of the large intestine in patients with irritable bowel syndrome (IBS) [58].

In an animal model (rats), the anti-secretory effect of SST via SST2 present on colonocytes was demonstrated [59]. In an in vitro model (HT-29cl.19A colonic cells) an inhibition of chloride secretion by SST was also described [60]. The SST analogue octreotide (OCT) also promotes mucin 2 (MUC2) expression and mucus secretion by human goblet-like cells (LS174T) via SST5 and suppresses Notch-Hes1 signaling [61]. It should be added that autonomic cells and some enterohormones (including SST) are also involved in the rhythm of cell proliferation in the GIT and liver and are subject to many circadian rhythms. Circadian disruption accelerates aging and promotes tumorigenesis in the GIT [62]. On the other hand, two hypothalamic hormones, i.e., growth hormone-releasing hormone (GHRH) and SST, are affected by the aging process, which in turn influences numerous age-related changes (including cancer) [63].

## 3. Somatostatin and the Histological Spectrum of CRC

In the histogenesis of colorectal NENs, some important cells are EECs/neuroendocrine cells (NCs), which are adjacent to colonic stem cells (SCs) in the SCs crypt niche [37]. Cooperation between CSCs and NCs is possible [37,38]. It is known that adenomatous polyposis coli (*APC*) mutation leads to the development of CRC, also through reduced maturation of aldehyde dehydrogenase-positive (ALDH+) SCs into progenitor NCs and reduced feedback by mature NCs [38].

Around 17 different types of NCs have been described in the GEP system, but the role of precursor cells and the biological mechanisms of GEP-NET formation are only partially understood [64]. In nonneoplastic and neoplastic GIT tissues and ENS structures, NCs express a panel of identical antigens that are used as NE markers. Their presence, even without hormone production, is sufficient to reveal NE differentiation [65].

Somatostatin is used as one of the markers of NE differentiation in CRC [32,34,66,67,68,69]. The number of NCs expressing specific markers (including SST) decreases in adenomas and carcinomas compared to normal colonic crypts [38]. Using the IHC method exocrine and NE differentiation markers, four groups of colon carcinomas were initially distinguished: (i) pure exocrine carcinomas; (ii) pure NE carcinomas; (iii) mixed exocrine and NE carcinomas; and (iv) exocrine carcinomas with sporadic NCs. The largest group consisted of mixed exocrine/NE cancers, while the second largest group showed only features of the exocrine phenotype [66]. The WHO classification (2000) distinguishes eight types of tumors of the colon and rectum [70]. However, the 2010 WHO classification redefines the concept of intraepithelial neoplasia and dysplasia and introduces certain changes in the nomenclature and diagnosis of CRC. It adds new CRC subtypes such as serrated adenocarcinoma, cribriform comedo type adenocarcinoma and micropapillary adenocarcinoma. The changes also concern the nomenclature and classification of NE tumors of the lower GIT. The new classification clarifies the grade of malignancy for mucinous and signet ring cell carcinomas (SRCC), taking into account the microsatellite instability (MIN) pathway, as those with a better (high MIN grade) and worse prognosis (low or no MIN grade). It also describes polypoid lesions and adenomas of the large intestine in more detail [71].

In the latest classification (2019), changes concern the nomenclature of serrated lesions of the colon, rectum and appendix. The preferred name is serrated lesions because they may be flat rather than polypoid and the association with a *BRAF* or *KRAS* mutation defines two distinct cancer pathways [72]. In this classification, special attention was also paid to NENs [72,73]. Their previous division was maintained [71,74] on neuroendocrine tumors (NETs) (grade (G) 1 and 2 tumors) and a new category was added—G3 NET and poorly differentiated NE carcinomas (NECs) (G3 neoplasms, including small-cell and large-cell types carcinomas). This division is based on molecular differences, mainly on the presence of mutations in multiple endocrine neoplasia 1 (*MEN1*), death-domain associated protein (*DAXX*) and ATP-dependent helicase ATRX (*ATRX*) (in well-differentiated NETs), tumor protein 53 (*TP53*) and retinoblastoma (*RB*) (in NECs). The grading criteria include mitotic rate (mitoses/2 mm^2^) and Ki-67 index (from below 3% to above 20%) [72]. The name of mixed adenoneuroendocrine carcinomas (MANECs) was also changed to mixed neuroendocrine-non-neuroendocrine neoplasm (MiNEN). In this tumor, both the NE and non-NE components are poorly differentiated, and the NE component has proliferation rates in the same range as other NECs [72,73,75]. The histological features of colorectal NETs and NECs are similar to those found in other organs. MiNENs of the large intestine consist of a poorly differentiated component and an adenocarcinoma component.

A conventional IHC biomarkers of the NE lineage in the NENs’ classification is SSTRs, next to insulinoma-associated protein 1 (INSM1), synaptophysin and chromogranin. The diagnosis of highly proliferative, well-differentiated NETs is obliged to search for biomarkers that can distinguish G3 NETs from NECs, including SSTRs [73,75]. A category including NE differentiation below 30% but above the level described for normal colonic epithelium (>1 cell/mm^2^) [76], >2% [77], and similar to that proposed by Jansson et al. [66], was not defined in the WHO classification from 2010 [71] or in the latest one from 2019 [72,73].

In total, 85–90% of CRC cases are adenocarcinomas of varying degrees of differentiation [78,79,80]. About 20% of them are poorly differentiated or undifferentiated cancers with a worse prognosis [71]. Colorectal NENs are rare types of CRCs, as are squamous cells, adenosquamous cells, spindle cells and undifferentiated carcinomas [80]. The presence of NCs is detected in ~8–85% of sporadic CRCs [33,65]. NE differentiation was also demonstrated in >50% of cases of hereditary non-polyposis CRC (HNPCC) [81]. The clinical significance of NE differentiation in sporadic CRC is controversial [65,69,76,82]. It has been shown that patients with multiple NCs in CRC had a significantly worse prognosis compared to patients without EECs [67,77,83]. NE differentiation was observed more often in small cell undifferentiated CRC, which correlated with tumor progression [77]. Other studies in CRC showed a better prognosis for 5-year survival the higher the detection rate of NCs and their hormonal products [69]. In a study of a large cohort (>1000 CRCs), it was found that patients with colorectal MANECs have significantly worse clinical outcomes than all patients with other subtypes of colorectal adenocarcinoma known from the latest WHO classification [84]. Mixed adenoneuroendocrine tumor (MANET) with dual differentiation (NE and adenoma) with each component accounting for at least 30% of the tumor was also distinguished [85]. MiNENs with a low-grade NET component can rarely occur in the background of idiopathic inflammation and are also present in patients with inflammatory bowel diseases (IBDs) [86]. Some studies indicate that CRCs with the NE carcinomas phenotype have a similar mutational profile to colorectal adenocarcinomas, and compared to MANECs, they have a higher rate of *APC* mutation. It is therefore likely that the cells leading to the growth of these tumors have an intestinal coinage [35].

It was also suggested that not the features of NE but the relationship with the low degree of tumor differentiation, lymph node metastases, distant metastases and other unfavorable features results in worse clinical outcomes. However, other studies deny the existence of a relationship between NE differentiation and the prognosis of CRC (reviewed in [65]). However, as shown by a retrospective study on a large group of patients with NETs (*n* = 64,971), using nationally representative data from the Surveillance, Epidemiology, and End Results (SEER) program in the USA, in the case of distant NETs, those in the colon had the worst median overall survival (OS) (4 months). Regardless of location, patients with NET G3 and G4 had a low OS of 8 months in the cecum and colon, significantly lower than in the small intestine and appendix (30–33 months) [87]. In the Chinese population, colonic NENs also had a worse 5-year survival (~67%) compared to rectal NENs (~88%), but tumor location and tumor size and pathological classification were independent prognostic factors [88].

## 4. Antitumor Effects of Somatostatin in CRC

There are direct and indirect antitumor effects of SST and SSAs in various cancers. The direct action occurs through SSTRs present on cancer cells and includes inhibition of mitogenic signals dependent on growth factors, induction of apoptosis and inhibition of cell cycle and/or tumor growth. The indirect effect consists of inhibiting the exocrine and endocrine secretion of growth factors or trophic hormones, e.g., epidermal growth factor (EGF), basic fibroblast growth factor (bFGF) and/or insulin-like growth factor 1/2 (IGF-1/2). Indirect actions also include immune modulating effects and inhibition of tumor angiogenesis [23,26,27,28,89,90].

### 4.1. Inhibition of Cell Proliferation

Depending on the SSTR subtype, different signal transduction pathways are involved in the antiproliferative effect of SST, which have been studied in more detail using various in vivo and in vitro research models [19,26].

#### 4.1.1. Evidence from In Vivo Studies

Higher circadian SST concentrations were observed both in ulcerative colitis (UC) [91], in patients with colorectal polyps and in CRC compared to healthy people. This suggests a protective effect of SST in precancerous alterations of the colon and CRC [40]. Other studies by these authors conducted on CRC patients do not seem to confirm the antitrophic effect of SST at the cellular and subcellular levels [41].

Many studies have demonstrated the tissue expression of SST (mRNA, protein) and characterized the cells producing SST in this type of tumor. Most studies localize this peptide both in EECs, where colocalization with other NPs is common, e.g., chromogranin A, serotonin, glucagon, bombesin, vasoactive intestinal peptide (VIP), and in cancer cells (reviewed in [92]). Amphocrine features of CRC cells have been demonstrated [67,93]. This may confirm the theory of the origin of EECs [34] or the development of this type of tumor from multipotential endodermal SCs [32,34]. Based on in vivo and in vitro studies, it has been shown that SST signaling controls the rate of NCs maturation as SCs mature along the NE cell lineage, which contributes to SC silencing and inhibition of cell proliferation [37]. Additionally, SST expression was demonstrated in the structures of the nervous system present in the tumor, where it also co-expressed with other NPs, e.g., protein gene product 9.5 (PGP 9.5), substance P (SP) and calcitonin gene-related peptide (CGRP) [68].

Typically, decreased SST expression was observed in CRC compared to normal colon tissues [94,95,96,97] or was not detected at all either at the protein [98] or mRNA [37] level. This was confirmed by studies on an animal model (male rats with 1,2-dimethylhydrazine dihydrochloride-induced colonic adenocarcinoma), which showed only a few SST-positive cells [99]. Low SST tissue expression suggests loss of the inhibitory role of SST in tumor growth. Other authors consider the decrease in SST expression and the increase in ectopic expression of other NPs as indicators of preneoplastic changes in the large intestine [96]. A reduction in cellular SST expression in CRC is correlated with poor grading and staging [95]. However, an increase in the number of highly differentiated NCs compared to the number of poorly differentiated NCs in CRC was also observed [69]. The latest bioinformatic analysis indicates six hub genes, including SST and SST2, that were significantly downregulated in colon adenocarcinoma compared to controls [100]. SST deficiency or abnormal function of its receptors would be risk factors for the development of CRC.

Administration of various SSAs resulted in a reduction in tissue expression of proliferative antigens, i.e., Ki-67 in 4/12 patients with rectal cancer (RC) [101] and proliferating cell nuclear antigen (PCNA) in 6/10 patients with CRC versus control [102]. Moreover, SSAs treatment reduced serum levels of GH and IGF-1 [103,104,105,106]. A decrease in the average percentage of cells in the S phase was also observed due to the simultaneous reduction in IGF-1 in the blood serum of these patients, but without changes in GH and EGF concentrations [106]. Another study using lanreotide (LAN) in advanced CRC did not confirm the antitumor activity of this SSA. Only higher doses of LAN seemed to maintain reduced IGF-1 concentrations in the blood serum of these patients [105].

The mechanisms of the antiproliferative effect of natural SST in CRC can be also associated with the abnormal expression of cyclins (D1, A, E) and cyclin-dependent kinases (CDK2 and CDK4) in CRC tissues. SST would regulate the stage of entry into the S phase of the cell cycle [107].

#### 4.1.2. Evidence from In Vitro and Animal Model Studies

Inhibition of proliferation, increase in apoptosis of CRC cells, inhibition of tumor growth in animals (nude mice bearing xenografts) and reduction in angiogenesis after the use of SSAs have been proven in numerous studies both in vitro and in vivo. The most common CRC cell lines tested for this purpose were, in descending order, HT-29, HCT-116, Caco-2, SW480 and SW620. All these lines are formed by cells with an epithelial phenotype derived from patients with colorectal adenocarcinoma. The most frequently represented work is performed on the HT-29 cell line which is derived from a human colon adenocarcinoma originating from a 44-year-old female [108]. HT-29 cells showed particularly high susceptibility to several modified octapeptide analogues of SST containing unnatural amino acids (AA) compared to other cell lines (MDA-MB-23, HepG2, HeLa and Lep-3). The most pronounced antiproliferative effects were demonstrated by the compound 4 C: Orn^5^ and α-aminoisobutyric acid (6) (Aib^6^) in these cells with the IC50 = 0.0199 μM [109].

HCT-116 and HT-29 represent CRC cell lines that correspond to the more (Dukes’ D) and less aggressive forms (Dukes’ C) of CRC, respectively [110,111]. HCT-116 cells were originally isolated from primary tumors derived from colon ascendens of a 48-year-old male [112]. Cells from these lines possessed different statuses of one of the most commonly mutated genes in CRC, i.e., *KRAS*. HT-29 cells which have microsatellite stable (MSS) status had wild-type (wt) *KRAS* and *PTEN*, but *BRAF*, *PIK3CA* and *TP53* mutations (R273H), whereas HCT-116 cells, which have MSI status, gained mutated *KRAS* and *PIK3CA* [110].

In HT-29 cells and nude mouse xenografts, an obvious inhibition of proliferation was demonstrated after SST/SSA treatment [109,113,114,115,116,117,118,119,120]. Some authors observed the antiproliferative effect of SST, but only in the presence of serum [114] or in a time and dose-dependent manner [109,118,119,120]. Differences in the intensity of the antiproliferative effect of two SSAs (Sandostatin and TT-232) on HT-29 cells were demonstrated. After treatment with TT-232, a 59 ± 6% decrease in the cancer cell number was observed, and after Sandostatin, only 21 ± 12% [116]. However, Keri et al. showed that TT-232 was effective in inhibiting tumor growth (up to 70% inhibition) in the case of transplanted animal tumors (including C26) and human tumor xenografts. In the case of HT-29 cells, the inhibition of proliferation also reached 72 ± 5% [121].

The antiproliferative effect of SSAs has also been described in other CRC cell lines, e.g., CX1 [113], LIM 1215, LIM 1863, LIM 2405, LIM 2412 [122], SW480 [123], SW620 [121,124], HCT-116 [125] and Caco-2 [119]. It was not observed in individual cases of the CRC cell lines tested, e.g., X56 [113], and rat colon cancer cells DHD/K12 [126]. Only a minimal effect was observed after administration of Sandostatin in the case of C170 and LIM 1215 cells [127]. No effect of SST on the invasive potential of murine colon adenocarcinoma cells 26L5 was observed [128].

OCT dose-dependent inhibition of proliferation and cell arrest in the G1 phase of the cell cycle were also observed in SW480 cells [129]. Szepeshazi et al. showed that another SST derivative, AN-238, inhibits the growth of experimental colon cancers (HT-29 and HCT-15 cells) that express SSTRs, regardless of their p53 status [130]. No significant differences in the intensity of cell proliferation were observed in mice injected with human colon cancer cells using triple therapy with OCT + galanin + serotonin versus 5-fluorouracil/leucovorin (5-FU/LV) [131] or compared to 5-FU/LV-irinotecan or 5FU/LV-oxaliplatin [132].

To further assess the direct antiproliferative mechanisms of SST, the presence of SSTRs (or specific binding sites) on cultured CRC cells was also examined as a condition for the action of SST/SSAs. In the case of HT-29 cells, binding sites for SST, bombesin and EGF [115], the existence of low-affinity SSTRs in such cells [133], high-affinity binding sites for SST [125] and functional SSTRs was shown [130]. Using the immunofluorescence technique (IF), the presence of receptor subtypes such as SST3/4/5 [134] and all types of SSTRs, including two isoforms of SST2 (SST2A and SST2B), was demonstrated. The presence of SST1/2/5 in HT-29 cells was demonstrated using RT-PCR [119]. In the case of Caco-2 cells, the presence of SST3/5 was demonstrated and in HCT-116 cells, SST2/3/5 [134].

Research by some authors showed that SST preferred SST3 and SST5 in their effects [120,134]. However, no functional SSTRs were detected on LoVo cells; hence, none of the SSAs used inhibited the proliferation of LoVo tumors [130]. LoVo cells are cells from a 56-year-old male, with Dukes’ C, with MSI status, with a KRAS mutation, but without mutations in other important genes of intestinal carcinogenesis (e.g., BRAF, PIK3CA, PTEN, TP53) [110].

It seems that greater antiproliferative effects occur when SSAs are used in combination with other antitumor agents. The use of a combination of OCT with interleukin 2 (IL-2) and interferon gamma (IFN-γ) gave a stronger antiproliferative effect on the growth of SW620 cells but not in SW480 cells [123]. Similarly, the use of Sandostatin together with 5-FU gave a stronger antiproliferative effect than the administration of Sandostatin alone in C170 and LIM 1215 cells [127]. These observations are confirmed by the study by Massari et al., who in the colon cancer cell line WiDr (identical with HT-29 cells), expressing a mutant p53 (mp53), showed that the SMS analogue has pro-apoptotic and antiproliferative effects, which can enhance the effect of 5-FU on human CRC cells expressing mp53 [135].

Recent studies also indicate better antiproliferative effects after using conjugated cetuximab (CTX)–OCT loaded onto Ca–alginate beads (CTX-OCT-Alg) compared to free drug. The studies were performed on three different cell lines, including CRC cells (HCT-116) [136]. The latest work by Fan et al. demonstrates a combined antitumor effect on cells with the presence of SSTRs, as well as a mouse model treated with thymidine kinase (TK) deleted vaccinia virus Tian Tan strain Guang9 (VG9/TK-) or VG9/(SST-14)_2_-human serum albumin (HSA). Fusion technology was used to extend the half-life of SST in the circulation by combining natural SST with a full-length HSA molecule. It was shown that VG9/(SST-14)_2_-HSA is more effective in prolonging the survival of all mice in both groups than VG9/TK-. However, the oncolytic activity of vaccinia viruses was not high enough in some cells, including HCT-116 cells, indicating that these cells were more resistant to the effects of the viruses. However, as a whole, these studies indicate that vaccinia VG9/(SST-14)_2_-HSA has oncolytic activity of the virus as well as anticancer activity [137].

#### 4.1.3. Mechanisms of the Antiproliferative Action of SST

Studies on the mechanisms of action of SST/SSAs in CRC cell lines or animal models confirm the involvement of known intracellular pathways that regulate secretory activity, inhibit cell proliferation and increase cell apoptosis [15,16,20,22,89,90].

The antiproliferative mechanisms of SST/SSAs in CRC are related to the regulation of protein phosphorylation on tyrosine residues, which is an important cell signaling mechanism [16]. The control of protein phosphorylation/dephosphorylation occurs through the combined actions of protein-tyrosine kinases (PTKs) and protein-tyrosine phosphatases (PTPs), respectively [138]. They are essential for cellular homeostasis and can lead to disruptions in various important cellular pathways, including cell proliferation and differentiation. It is also known that >80% of all oncogenes encode PTKs, and PTPs that can reverse the action of PTKs play an important role as tumor suppressors [24].

As research by Keri et al. showed, a structural derivative of SST, with a five-residue ring (D-Phe-Cys-Tyr-D-Trp-Lys-Cys-Thr-NH2) called TT-232 inhibited the tyrosine kinase activity of some human carcinomas cell lines of the colon, and this inhibition correlated well with the antiproliferative effect but did not correlate with GH release inhibition [139,140]. Subsequent studies by these authors confirmed strong inhibition of tyrosine kinase activity (75%) after long-term incubation (24 h) with TT-232 in SW620 cell culture. Moreover, they showed that this effect correlated well with the inhibition of proliferation and the effect of inducing cell apoptosis. In addition, this study demonstrated the antiproliferative effect of TT-232 also on Colo205 cells (proliferation inhibition > 50%) and in an animal model (tumor growth inhibition ~70%) [121]. Other studies by the same group confirmed previous observations (strong antiproliferative effect of SW620 cells after TT-232) and additionally showed a rapid and sustained (5–30 min) increase in PTP activity [124]. It should be added that there are approximately 100 PTPs in the genome, approximately equivalent to the number of tyrosine kinases [141]. TT-232-induced PTP activation may therefore be an important early step in the signaling pathway in inhibiting cell proliferation in CRC [124]. Modulation of the activity of various PTPs is one of the intracellular pathways responsible for inhibiting cell growth also by OCT [119]. SST-stimulated PTP activity shares biochemical features with SHP1 and SHP2 phosphatases. These phosphatases belong to a family of cytosolic PTPs that contain motifs called src homology 2 (SH2) domains and are involved in protein–protein interactions through their association with specific phosphotyrosine residues [142]. The PTP family also includes density-enhanced phosphatase-1 (DEP-1) (humans)/PTPη (in rats). All are intracellular effectors of SSTRs [16].

Another mechanism of the antiproliferative effect of SST (also involving PTPs) in CRC cells (Caco-2, HT-29 and HCT-116) is the inhibition of cyclooxygenase-2 (COX-2) and prostaglandin E2 (PGE2). In Caco-2 cells, SST-14 has been shown to inhibit basal COX-2 expression, PGE2 production, DNA synthesis and cell growth. The inhibitory effect of COX-2 expression and function occurs through the activation of SST3 or SST5. Therefore, SST may oppose proliferative stimuli by reducing COX-2 expression driven by a negative regulation of protein kinase C-dependent mitogen-activated protein kinase (MAPK) and AKT activation. The attenuation of constitutive COX-2 expression by SST in CRC cells via SST3/5 is expected to occur through activation of PTP, which leads to the inhibition of MAPK signaling and is the main mechanism for inhibiting the growth of CRC cells [134].

Uncontrolled growth of CRC cells is also enabled by the activation of human telomerase reverse transcriptase (hTERT), a catalytic component of the telomerase complex. Telomerase, which maintains telomere length and maintains the cell’s replicative potential, is activated during the adenoma–carcinoma sequence and its activity increases during tumor progression. It is believed that telomere shortening plays a role in the early stages of colorectal carcinogenesis, resulting in chromosome instability [143]. The telomerase signaling was also examined in HT-29 and Caco-2 cells after OCT treatment. Increased telomerase activity in HT-29 cells cultured in the absence of serum and in the presence of 10% fetal bovine serum (FBS) was demonstrated. However, in Caco-2 cells, a decrease in telomerase activity was observed in cells cultured without serum and an increase in the presence of TBS. The authors speculate that OCT may inhibit cell proliferation selectively in Caco-2 cells by reducing telomerase activity, whereas in HT-29 cells it appears to inhibit cell proliferation through different molecular pathways [119].

An important mechanism of SST action is also the regulation of the distribution and expression of p86 Ku protein (Ku86), the regulatory subunit of DNA-dependent kinase and the SST binding site. Ku86 has been shown to behave as a specific nuclear receptor for SST and regulate p53 expression and apoptosis [121,144,145]. Quite early, it was shown that Ku86 modulates in vitro dephosphorylation of p34CDK2–phosphorylated histone H1 by phosphoprotein phosphatase 2A (PP2A) [144]. PP2A is one of the phosphoprotein phosphatases (PPPs), the largest family of phosphatases, and dephosphorylates hundreds of substrates involved in the cell cycle, regulating almost all major pathways (including MAPK and Wnt/β–catenin pathways) and cell cycle checkpoints [141]. These findings suggest that Ku86, as a nuclear SST receptor, may mediate negative control of cell cycle regulation by SST [144]. This is confirmed by other studies [116,145]. In addition to the quantitatively differentiated antiproliferative effect of Sandostatin (smaller) and TT-232 (larger) in HT-29 cells, the involvement of SST in the translocation of Ku86 from the cytosol to the cell nucleus was demonstrated [116]. However, studies with SST treatment of Caco-2 cells showed inhibition of cell growth while modulating the activation of the Ku70/86 heterodimer by SST. After SST treatment, an increase in Ku86 mRNA levels was observed in the cell nucleus. These studies confirm the hypothesis that SST controls cell cycle progression and DNA repair through a signaling pathway involving the regulation of Ku86 levels and Ku70/86 activity in the cell nucleus [145]. Further studies on Caco-2 cells showed that SST increases the binding between Ku70 and Ku86, induces an antiproliferative effect after 24 h and restores apoptosis in cells [146]. In turn, a new mechanism of cellular adaptation as a defense system against severe genomic stress caused by the functional loss of Ku70 has recently been discovered. Conditional deletion of *XRCC6* (the gene encoding Ku70) has been shown to promote an adaptive, opportunistic transition to a parasitic lifestyle of HCT-116 cells at the expense of continuous host cell exploitation [147].

In the SW480 cells model, it was shown that OCT can inhibit the growth of human colonic cancer cells also by inhibiting the Wnt/β–catenin signaling pathway [129]. They also showed correlations of reduced tumor growth with clinical tumor biomarkers after the use of SSAs. A 13% decrease in carcinoembryonic antigen (CEA) level was observed in LoVo cells after treatment with SMS 201.995. Similarly, in xenografts, a correlation was detected between a decrease in tumor growth and a reduced serum level of CEA. The CEA concentration therefore reflected the number of cells in vitro and the size of the tumor in vivo in response to treatment with this type of SSA [148].

### 4.2. Pro-Apoptotic Effects of Somatostatin

#### 4.2.1. Evidence from In Vivo Studies

There are few studies on the influence of SST on the apoptosis process in CRC in vivo. Mao et al. showed higher tissue expression of the pro-apoptotic protein Bax and lower expression of the anti-apoptotic protein Bcl-2 in CRC groups with high and moderate SST expression compared to the low expression group [149]. Similarly, apoptosis rates determined by the expression of Fas protein and two caspases (3 and 8) were higher in the groups of CRC patients with high and moderate SST expression compared to the low expression group [150].

#### 4.2.2. Evidence from In Vitro and Animal Model Studies

The pro-apoptotic effects of SST/SSAs in vitro or in animal models were most often demonstrated in parallel with their antiproliferative effect or tumor growth inhibitory effect [97,121,125,129,135,151,152]. In HT-29 cells, after using TT-232, a 7-fold increase in the number of cells in apoptosis was demonstrated. However, in SW620 cells, a positive correlation was obtained between the increase in apoptosis and the antiproliferative effect in long-term incubation (24 h) with this SSA. The studies were confirmed in a mouse model, where a 70% inhibition of tumor growth was achieved in the transplanted Colon 26 tumor. The apoptosis-inducing effect was independent of p53, as there was no significant effect of TT-232 on the translocation of Ku86 from the cytosol to the cell nucleus [121]. However, in the murine transplantable Colon 38 cancer model, it was shown that when administered separately, both SMS and melatonin (Mel) significantly reduced the index of cell proliferation (labeling index, LI) and increased the apoptotic index (AI). However, no additive effect of SMS and Mel on cell proliferation or apoptosis was observed. The imbalance between the processes of proliferation and apoptosis has changed in favor of cell death [151]. Massari et al. showed the pro-apoptotic activity of the SMS 201.995, which was demonstrated together with the inhibition of cell proliferation in the WiDr cell line (identical to HT-29) [135]. Hohla et al. showed an increase in the number of apoptotic cells and inhibition of the proliferation of HTC-116 and P388/R84 cells in the S/G2 phase [125]. Inhibition of tumor growth and induction of apoptosis also depend on the OCT dose, as demonstrated in SW480 cells [129]. The same cells showed an increase in apoptosis mediated by SST2 and SST5 [152]. However, the administration of OCT to Caco-2 cell cultures increased in the percentage of cells with apoptosis, increased DNA fragmentation (Sub-G1 population) and decreased proliferation [97].

El-Salhy et al. in their work using a mouse model with implanted rat or human colonic adenocarcinoma cells and the administration of triple therapy (OCT + galanin + serotonin) obtained an increase in apoptosis and a decrease in the number of blood vessels, without a decrease in the proliferative index (PI) compared to control [153]. When the effects of triple therapy (including OCT) were compared with LV/5-FU therapy, a decrease in PI and the number of blood vessels was demonstrated and an increase in AI in mice treated with both LV/FU-triple therapy and with triple therapy only as compared with LV/FU-treated mice [154]. Another two studies on xenografts confirmed an increase in AI and a decrease in the number of tumor blood vessels compared to the control after the use of triple therapy (including OCT) [155] and an additional decrease in PI [156]. The reduction in tumor volume and weight after triple therapy appeared to be due to low proliferation and increased apoptosis and decreased tumor vascularity [155]. In subsequent studies by these authors, no significant differences were observed in relation to cellular apoptosis in mice injected with human colon cancer cells after the use of 5-FU/LV [131], 5-FU/LV-irinotecan or 5FU/LV-oxaliplatin [157] compared with triple therapy.

Interesting research on the apoptosis process concerns the use of a combined preparation consisting of four NPs (including SST) called DRF 7295. This peptide caused an increase in p53 levels, downregulation of Bcl-2 levels in Colo205 cells and induction of active caspase-3 in HT-29 cells [158,159].

#### 4.2.3. Mechanisms of the Pro-Apoptotic Action of SST

The study of the pro-apoptotic activities of SST/SSAs in CRC in vitro resulted in the demonstration of certain mechanisms of apoptosis induction in cancer cells. The work of Keri et al. demonstrated strong inhibition of tyrosine kinase activity (75%) after long-term incubation (24 h) with TT-232 in SW620 cells, which correlated with the apoptosis-inducing effect [121]. An important mechanism of action of SST in modulating apoptosis in Caco-2 cells was determined via the interaction between Ku70, a nuclear isoform of clusterin (nCLU) and Bax. Increased levels of nCLU and significant induction of Bax were observed after SST treatment. The 55 kD nCLU is important in the apoptosis process and acts as a chaperone in DNA repair by binding Ku70. A 10-fold increase in the interaction between Ku70 and nCLU was observed after SST treatment compared to untreated cells. However, after 24 h of SST treatment, Bax was released and nCLU and Ku70 colocalized in the cell membrane and nucleus [146].

Another mechanism of OCT inducing apoptosis, demonstrated in SW480 cells, might be the negative regulation of the Wnt/β–catenin signaling pathway via SST2 and SST5. After OCT treatment of cancer cells, accumulation of β-catenin in plasmalemma, inhibition of T-cell factor-dependent transcription, downregulated Wnt target genes (*cyclin D1* and *c-Myc*) and mediation of activation of glycogen synthase kinase 3β (GSK-3β) [152] were observed.

**Figure 1:**
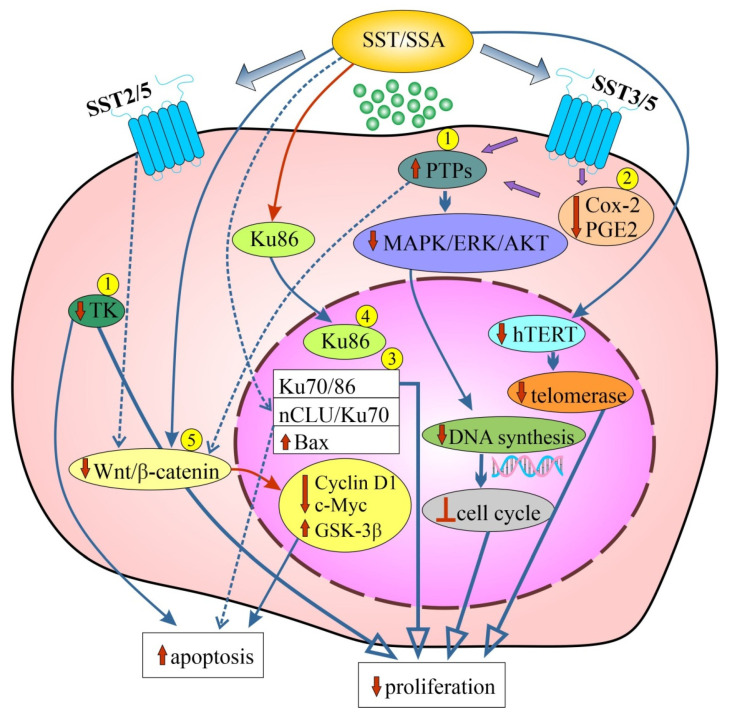
Schematic representation of the effects of SST related to inhibition of cell proliferation and stimulation of apoptosis in various colorectal cancer (CRC) cell lines (1–5) and/or in CRC animal models using SSAs (please refer to the main text for more details). Dashed lines with arrows indicate regulation/modulation of other signaling molecules/pathways. [↓/↑-reduced/increased expression/activity; Ʇ: inhibition, blockade; 1: SW620 cells; 2: Caco-2 cells; HT-29 cells; HCT-116 cells; 3: Caco-2 cells; 4: HT-29 cells; 5: SW480 cells; Cox: 2-cyclooxygenase-2; GSK: 3β-glycogen synthase kinase 3β; hTERT: human telomerase reverse transcriptase; MAPK/ERK/AKT: mitogen-activated protein kinase/extracellular signal regulated kinase/serine/threonine-protein kinase (protein kinase B); nCLU: nuclear clusterin; PGE2: prostaglandin E2; PTPs: protein-tyrosine phosphatases; SSA: synthetic somatostatin analogue; SST: somatostatin; SST2/3/5: somatostatin receptors 2/3/5; TK: tyrosine kinase; Wnt/β catenin: wingless + integrated or int-1/beta-catenin].

A summary of the mechanisms of action of SST to inhibit CRC cell proliferation and induce cell apoptosis, as well as inhibiting angiogenesis using SSAs, is shown in Table 1 and Figure 1.

Various SSAs have been used in studies on the antitumor activity of SSTs, which also acted as agonists of the respective SSTRs. Synthetic derivatives of SST have similar activity to native SST but with a longer half-life [89]. The tables in this review contain the original names of the types of SSAs used, provided by the authors. Scientific publications cited in the tables are arranged according to the year of published research.

The overwhelming number of studies used OCT (SMS 201-995, Sandostatin), which has an affinity for SST2 and SST5 and a structural SST derivative termed TT-232, a peptide agonist for SST1/SST4 [23,89]. SSAs were used as independent SSAs and/or in complexes with other drugs (Table 1). Detailed characteristics of SSAs are presented in Section 6.1.

### 4.3. Antiangiogenic Effects of Somatostatin

The antiangiogenic characteristics of SST/SSAs are at least partially related to the antiproliferative activities of these peptides [160]. Direct antiangiogenic effects are observed in cancers where SST2 and SST3 predominate, present on tumor cells and/or proliferating vascular endothelial cells (ECs) [21,25,28,161,162]. The antiangiogenic effect of SST may also be indirect through inhibition of the secretion of vascular growth factors (e.g., VEGF and bFGF), reduction in monocyte chemotaxis or through its immunomodulatory effects [28,161,163].

#### 4.3.1. Evidence from In Vivo Studies

Research by Reubi et al. suggests a direct role of SST in angiogenesis and the regulation of hemodynamic tumor–host interactions involving the TME via SSTRs located in the wall of blood vessels within the tumor (including colonic adenocarcinoma). Overexpression of SSTRs (presumably SST2) was demonstrated in peritumoral vessels, mainly in the muscular layer of peritumoral veins, with high affinity for SST-14, SST-28 and OCT in human primary CRC. This expression was independent of receptor expression in tumor cells [164].

Regarding the indirect role of SST in angiogenesis, a significant reduction in both tissue expression and serum values of VEGF was observed in CRC patients (*n* = 35) after OCT treatment before surgery. This is the only in vivo study on the role of SST in angiogenesis in sporadic CRC [165]. However, a decrease in VEGF concentration and an increase in VEGF receptor type 1 (VEGFR-1) concentration were observed with first-generation long-acting (LA) SSAs (LAN, OCT) in patients with NENs (*n* = 56). The tested angiogenesis markers (VEGF and VEGFR-1) seem to have limited usefulness in assessing the effectiveness of SSA treatment in NENs, but they are useful in the differential diagnosis of NENs and healthy people [166]. The antiangiogenic effects of OCT were also demonstrated by studies in nude mice xenografted with NE human RC. Both NCs and vascular endothelium within the tumor expressed SST. The use of OCT resulted in a decrease in plasma levels of VEGF and bFGF, an increase in NE carcinoma apoptosis, a decrease in the number of microvessels and inhibition of angiogenesis in the tumor [167].

**Figure 2:**
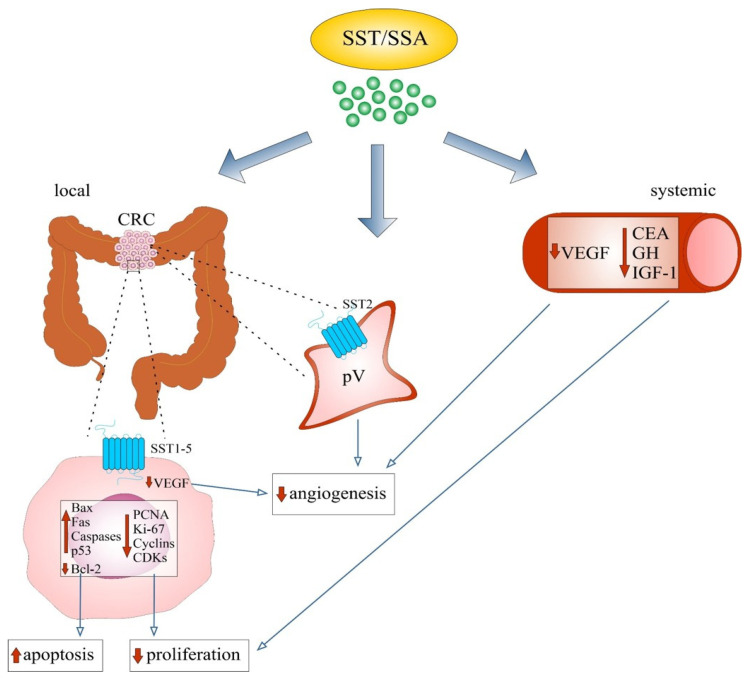
Schematic representation showing local and systemic effects of somatostatin (SST) and its synthetic analogue (SSA) associated with inhibition of cell proliferation and stimulation of apoptosis, as well as inhibition of angiogenesis in colorectal cancer (CRC) in vivo (please refer to the main text for more details). [↓/↑: reduced/increased expression/activity; CDK: cyclin-dependent kinases; CEA: carcinoembryonic antigen; GH: growth hormone; IGF-1: insulin-like growth factor 1; PCNA: proliferating cell nuclear antigen; pV: peritumoral vein; SST1-5: somatostatin receptors 1–5; VEGF: vascular endothelial growth factor].

A summary of the mechanisms of action of SST leading to inhibition of cellular proliferation and increased apoptosis, as well as inhibition of angiogenesis in CRC in vivo, is presented in Table 2 and Figure 2.

#### 4.3.2. Evidence from In Vitro and Animal Model Studies

An animal model (dogs) showed a dose-dependent decrease in intestinal blood flow, a decrease in capillary surface area and intestinal oxygen consumption and a significant increase in intestinal motor activity. SST acted on the smooth muscles of both arterioles and precapillary sphincters, which resulted in strong vasoconstriction in the intestinal microcirculation [168]. Moreover, SST has different effects on the arterial and venous walls, as well as different effects on large and small arteries. The main reaction in the arteries is their vasodilation [169]. SST/SSAs also inhibited the proliferation of rat aortic vascular smooth muscle cells (VSMCs) by activating human SST5-like receptors [170]. There was a concomitant promotion of ECs proliferation [170,171] and reducing neointimal formation [171].

In various in vitro and in vivo models, OCT acetate (SMS 201-995) has been shown to reduce the proliferation of human umbilical vein EC (HUV-EC-C; HUVEC) and the vascular network of the chick chorioallantoic membrane. OCT was therefore considered an inhibitor of angiogenesis [160]. Normal arteries and veins and those with atherosclerotic lesions show high levels of SST1 and no expression of SST3 and SST5. Interestingly, the presence of SST1 affected only ECs but not VSMCs. Exposure of ECs to SST or an SST1-specific agonist resulted in changes in the actin cytoskeleton [172]. In turn, other studies showed that *SST2* was expressed in proliferating angiogenic buds of human vascular endothelium [173]. However, the research by Florio et al. showed that SST directly affects the proliferation of various EC lines by blocking growth factor-stimulated MAPK (MEK) and endothelial nitric oxide synthase (eNOS) activity [162].

Hypoxia leads to increased angiogenic potential by increasing VEGF expression and secretion. Both SST and SST1 agonist (CH-275), acting on hypoxic HUVECs, inhibited VEGF expression by regulating the activity of signal transducers and activators of transcription 3 (STAT3) and hypoxia-inducible factor 1 (HIF-1) [174]. However, other studies using the coculture of SST-producing endocrine cell line STC-1 and HUVECs showed a significant decrease in EC proliferation, a slight increase in motility and an increased capacity of tubule formation. SST would antagonize the effects of VEGF on EC proliferation but not on EC sprouting. However, it appears that the antiangiogenic effects of SST and OCT are probably effectively counterbalanced in the TME by the simultaneous release of proangiogenic factors such as VEGF [175].

A different perspective on the role of SST in angiogenesis was provided by a recent study [176]. In HUVECs under quiescent conditions, the expression of mainly SST1 and SST5 was confirmed. It has been shown that SST (primarily SST-14) prepares HUVECs for thrombin-induced hyperpermeability mainly through activation of MEK/ERK signaling. SST activated PI3K/AKT and p42/44 MAPK pathway by phosphorylation, i.e., promoted HUVECs proliferation and angiogenesis in vitro. Treatment of these cells with SST enhanced VEGF-induced angiogenesis. The authors explain these differences by the lack of SST2 and SST3 in HUVECs under the culture conditions used [176] and those typical for tumor angiogenesis [21,25].

El-Salhy et al. in their studies using an animal model and the administration of SSAs in various combinations with other NPs and chemotherapeutics indicate a decrease in the number of tumor blood vessels in connection with an increase in AI [153,154,155] or a decrease in PI [156] in CRC compared to the control. Antiangiogenic features were also demonstrated for the DRF 7295, which is a combination of SST with three other NPs (VIP, bombesin and SP). Concerning angiogenesis, this compound caused inhibition of VEGF secretion in HT-29 cells, as well as inhibition of capillary tube-like formation in ECs [159].

### 4.4. Anti-Inflammatory Effects of Somatostatin

CRC is considered an example of cancer closely related to chronic inflammation, which may occur at the earliest stages of cancer development [177,178]. Chronic inflammation promotes tumorigenesis; on the other hand, chronic activation of the mucosal immune system may, under certain conditions, protect colon cells against dysplasia [177]. The role of tumor cell interactions with the TME, composed of immune cells, stromal cells and the gut microbiota, to suppress or evade the immune response is increasingly emphasized. Attempts are being made to explain how inflammatory processes shape the immunological background of CRC [11]. Recently, the first analysis of the relationship between the expression of the SST receptor family, tumor immune infiltration and prognosis in CRC was performed. A strong relationship between the expression of SSTRs and immune cell infiltration has been demonstrated [179].

Somatostatin is considered an anti-inflammatory peptide and plays a significant role in mucosal immunity. The immunomodulatory role of SST in various research models is documented in papers from the 1990s and discussed in other reviews [180,181]. In a mouse model, intestinal inflammation was shown to increase SST mRNA in *SST2* knockout mice compared to wt mice, suggesting that SST mediates inflammation also in *SST2* null mice [56].

The mechanisms of immune control via the SST system in the adenoma–CRC sequence are, so far, poorly understood [25,182,183]. It seems that colon epithelial cells, to defend themselves against pathogens and the development of inflammation, produce, on the one hand, several pro-inflammatory cytokines and chemokines, and, on the other hand, some anti-inflammatory factors, including SST. Moreover, NPs (including SST) have been shown to directly induce the secretion of cytokines (e.g., IL-2, IFN-γ, IL-4 and IL-10) by T cells to regulate the intestinal immune barrier. NPs drive different populations of Th1 and Th2 lymphocytes to a forbidden secretion of Th2 cytokine from T and Th1 cell lines and vice versa [184]. SST and SSAs inhibit the expression of pro-inflammatory cytokines such as INF-γ [185] and tumor necrosis factor alpha (TNF-α) [186]. OCT increases the expression of TNF receptors 1 and 2 and enhances apoptosis of Kupffer cells [186].

Pioneering studies of the toponome in CRC mucosa have identified as many as 1930 motifs that differentiate normal human mucosa from the mucosa of CRC and have improved our understanding of the role of the adaptive immune system in CRC. In this tumor, an increased number of activated and cytotoxic T lymphocytes was observed, while the number of CD4(+)CD25(+) T lymphocytes was reduced and they were not activated, except for ulcerative colitis (UC). Additionally, the number of activated T lymphocytes with the (HLA)-DR(+) phenotype is increased, indicating altered antigen presentation. The expression of CD3(+)CD29(+) and the assembly of the lymphocyte function-associated antigen-1 (LFA-1) and LFA-3 receptors are altered differently, indicating a different regulation of T cell adhesion in this tumor. Increased numbers of natural killer (NK) and CD44(+) cells were also identified in CRC mucosa. The regulator of apoptosis in these cell populations would be nuclear factor-kappaB (NF-κB). Overall, CRC has been shown to induce a strong modification of protein expression profiles in lamina propria [187].

#### 4.4.1. Evidence from In Vivo Studies

The anti-inflammatory effects of SST and SSAs in vivo have been reported mainly in IBD, but the results are divergent [180,181,188]. IBD as a multifactorial chronic inflammatory condition includes UC, Crohn’s disease (CD) and microscopic colitis. The risk of CRC in IBD increases, among others, with the duration of IBD and the extent/degree of colitis [189]. Close relationships were shown between the circulating products of EECs and the course of IBD [190]. A decrease in the mucosal content of SST has been observed in these diseases, which seems to be the result of the inflammatory process (reduction in the number of EECs) rather than the cause of dysplasia and the gradual development of CRC [191]. An inverse relationship has been demonstrated between the amount of SST-containing cells and the grade of inflammation in CD [192]. Furthermore, a reduction in the number of SST-immunoreactive nerve fibers was observed in IBD. Changes in the perivascular nerves may be responsible for the congestion and ulcerations characteristic of these diseases [193]. Immunoreactive SST levels were also compared in patients with various GIT diseases. A particularly high increase in postprandial SST levels occurred in patients with active UC (176 ± 17 pg/mL) [194].

The pathogenesis of abnormal bowel function in CD may involve immune-mediated changes in enterochromaffin cell (EC) secretion of 5-HT. IL-1β and bacterial products (*E. coli* LPS) are more potent stimulators of 5-HT release in Crohn’s ECs compared to normal ECs. The mechanism of this secretion is via NF-κB and MAPK phosphorylation and can be inhibited by an IL-1β receptor antagonist and a Toll-like receptor 4 (TLR4) antagonist as well as an SST analogue, LAN [195].

#### 4.4.2. Evidence from In Vitro and Animal Model Studies

After treatment with SST-14 and OCT of cultured CRC cells (HT-29 and Caco-2), significant inhibition (>90%) of the secretion of pro-inflammatory cytokines (IL-8 and IL-1β) was demonstrated. Importantly, SST inhibited both the spontaneous secretion of IL-8 and IL-1β from intestinal cells, as well as the secretion initiated by bacterial invasion (*Salmonella* type D) or TNF-α stimulation. The inhibitory effect was dose-dependent, with maximum at a physiological concentration (3 × 10^−9^ M). Effects inhibiting the secretion of inflammatory mediators were observed at the level of transcription (decrease in mRNA) and with the participation of SSTRs (SST2/SST5 in Caco-2 cells and SST5 in HT-29 cells) [196].

Direct evidence for the inhibitory role of SST in the inflammatory process in the large intestine was obtained in animal models of colitis. Differences in SST expression have been demonstrated depending on the colitis model, as well as between animal colitis and IBD in humans. In trinitrobenzene sulfonic acid (TNBS)-induced colitis, an increase in SST-producing cells in the colon compared to normal tissue and a correlation of the density of SST-positive cells with the number of inflammatory cells (macrophages/monocytes and mast cells) were observed [197]. In TNBS-induced colitis and OCT administration, a reduction in TNF-α expression and inducible NOS (iNOS) activity was observed. OCT administration also reduced the production of IL-1β and IFN-γ in the colon [198]. After OCT treatment of rats with acetic acid-induced colitis, significant reductions in platelet activating factor activity, serum leukotriene B4 and VIP concentrations were observed. However, the mechanism of this action of OCT has not been determined [199]. Using dextran sodium sulfate (DSS)-induced colitis, it showed a lower density of SST-positive cells versus the control group, as in IBD patients. Changes in all EECs were accompanied by an increase in the densities of mucosal leukocytes, T and B lymphocytes, macrophages/monocytes and mast cells versus control. Regardless of the IBD model, these studies confirm the interactions between peptides produced by EECs (including SST) and immune cells in IBD [200].

Research indicates that colon infection and inflammation may lead to changes in the function of intercellular junctions, especially the tight junctions (TJs). It has also been shown that SST plays an important role in protecting the intestinal barrier by modulating the expression of TJ proteins. It appears that conserving intestinal epithelial TJ barrier integrity is a feasible and attractive approach to manage IBD [9,201,202,203]. In Caco-2 cells, SST significantly increased the expression of occludin and zonula occludens-1 (ZO-1) and inhibited the redistribution of TJ proteins induced by lipopolysaccharide (LPS) stimulation. The mechanism of the protective effect of SST against TJ breakdown in this study model was the reduction in ERK1/2 phosphorylation and the suppression of SST5 activation, both LPS-induced events. The authors suggest that SST5 may play an important role in intestinal barrier dysfunction (induced by LPS) and mediate the beneficial effect of SST on TJ damage in cancer cells [201].

In a mouse model with *Citrobacter rodentium*-induced colitis, an increase in the expression of occludin-1 and occludin-3 was observed after OCT administration compared to untreated mice. In turn, in the DSS-induced colitis model, OCT administration significantly increased the expression of only claudin-3 compared to the control. These results were also verified in Caco-2 cells by exposing them to enteropathogenic *Escherichia coli* (*E. coli*) and TNF-α. These were consistent with in vivo studies [202]. Similarly, in a mouse model with DSS-induced colitis, OCT effectively alleviated the progression of the disease, restored the structure and function of the intestinal barrier in the colon and also stimulated the expression of claudin-4. A similar increase in claudin-4 expression was observed in an in vitro model (Caco-2 cells treated with TNF-α). Increasing the expression of claudin-4 would occur by activating SST5 and suppressing ERK1/2 pathways [203].

Recent studies confirm the critical role of the SST5/myosin light chain kinase/myosin light chain (SST5/NF-κB/MLCK/MLC) signaling pathway in the mechanism of the protective effect of SST on the intestinal barrier. In other words, SST5 activation by its special agonist L817,818 protects the mechanical function of the intestinal barrier by increasing the expression of claudin-4 and ZO-1 via NF-κB/MLCK/MLC signaling [9].

**Figure 3:**
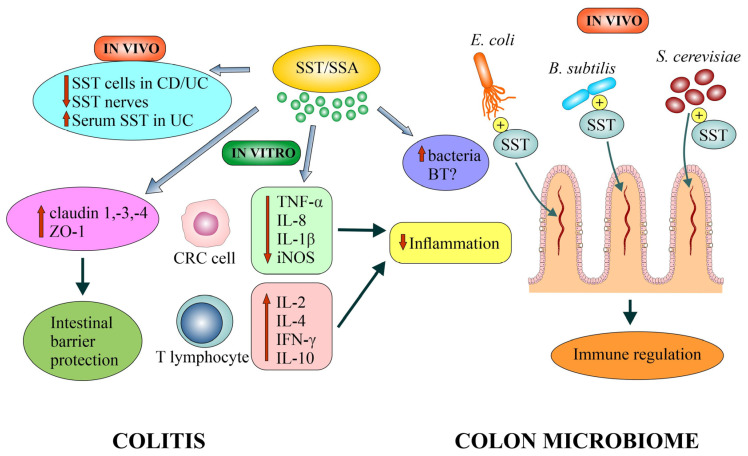
Schematic representation showing local and systemic effects of SST and SSAs associated with inhibition of inflammation in various in vitro models of colitis, as well as under in vivo settings (please refer to the main text for more details). [↓/↑: reduced/increased expression/activity; *B. subtilis*: *Bacillus subtilis*; BT: bacterial translocation; CD: Crohn’s disease; CRC: colorectal cancer; *E. coli*: *Escherichia coli*; IFN-γ: interferon gamma; IL-1β/2/4/8/10: interleukin 1β/2/4/8/10; iNOS: inducible nitric oxide synthase; *S. cerevisiae*: *Saccharomyces cerevisiae*; SSAs: somatostatin analogues; SST: somatostatin; TNF-α: tumor necrosis factor alpha; UC: ulcerative colitis; ZO-1: zonula occludens-1].

Interactions between NPs/amines (including SST) present in the GIT and the intestinal microbiota are increasingly considered to be crucial in the pathophysiology of IBD [188,190,204] (Figure 3).

A summary of selected mechanisms of the anti-inflammatory activity of SST in various models of colitis is presented in Table 3 and Figure 3.

### 4.5. Somatostatin and Colorectal Cancer’s Colon Microbiome (Microbiota)

In colorectal carcinogenesis, there is a close correlation between the tumor genome, TME and the host’s gut microbiome [205]. The intratumoral microbiota plays a special role in CRC as an important component of the TME. The microbiota participates in tumor formation and progression. It works through several mechanisms. It causes direct neoplastic transformation through toxic metabolites or induction of inflammation. The microbiota changes intestinal bacterial biofilms and disrupts TME homeostasis and the immune response. It may cause immunosuppression and influence the metabolism of drugs [205,206]. Intratumoral microorganisms are more common in mucosal cancers, including CRC [207]. There are significant differences in the structure and function of the proximal–distal sections of the large intestine in terms of histology, clinical features, genetics, immune system, *APC* mutant alleles or protein expression bias. Leading theories linking gut microbial dysbiosis with CRC are discussed in [204,208].

The human intestinal microbiota comprises trillions of microbes, the majority of which reside in the distal ileum and colon, on the surface of the mucosa [8,182,209]. Colitis, intestinal microbiota and CRC form a kind of “infernal triangle” [182,210]. Although the role of inflammatory cells and inflammatory mediators in the etiopathogenesis of colitis-associated CRC has been known, the role of the microbiota has not yet been fully evaluated [182]. The mouse model indicates that colitis facilitates the expansion of microbes that can promote CRC and create this “infernal triangle.” An animal model has shown that inflammation modifies the composition of the intestinal microbiota in IL-10-deficient (IL-10^(−/−)^) mice susceptible to colitis. Monocolonization with the commensal *E. coli* NC101 promoted invasive cancer in IL-10^(−/−)^ mice treated with the colo-specific carcinogen azoxymethane (AOM). Removal of the genotoxic polyketide synthase (pks) island from *E. coli* reduced tumor proliferation and invasion in AOM/IL-10^(−/−)^ mice, without affecting intestinal inflammation. Mucosa-associated pks(+) *E. coli* have been found in a high percentage of patients with IBD and CRC. This suggests that colitis may promote carcinogenesis by changing the microbial composition and inducing the expansion of microorganisms with genotoxic features [182].

No single microorganism has been identified as a risk factor for CRC. However, the development of adenoma and CRC may be influenced by a decrease in the number of protective bacteria, e.g., *Faecalibacterium prausnitzii* (*F. prausnitzii*) and members of the *Lachnospiraceae* family, as well as an increase in the number of certain bacteria (e.g., *Proteobacteria*, *Fusobacterium*, *Porphyromonas* and *Klebsiella*) and age-related changes in the microbiota (so-called pro-inflammatory intestinal phenotype) [8,204,207,211,212,213].

In the etiology of CRC, particularly *Fusobacterium nucleatum* (*F. nucleatum*), enterotoxigenic *Bacteroides fragilis*, *E. coli* and butyrate-producing bacteria are taken into account [8]. In 2016, it was discovered that a bacterium originally found in the oral cavity, *F. nucleatum*, can colonize CRC through hematogenous spread. The microbial protein lectin Fap2 plays an important role in the colonization process, attaching to the metastase host factor, D-galactose-β(1–3)-N-acetyl-D-galactosamine (Gal-GalNAc), which is overexpressed in the CRC [214]. Some bacteria may contribute to the progression of CRC by helping cancer cells evade the immune response by suppressing immune cell function, creating a pro-inflammatory environment or influencing the autophagy process [215]. In addition to the pathogens already mentioned, risk factors for the development of CRC include *Streptococcus bovis*, *Enterococcus faecalis* and *Peptostreptococcus anaerobius* [216].

Mechanisms related to the CRC microbiota have been described, including inflammation, pathogenic bacteria and their virulence factors, genotoxins, oxidative stress, bacterial metabolites and biofilm [216]. One of the genotoxins, i.e., colibactin produced by *E. coli*, is encoded by a conserved genomic island, “pks island”. This genotoxin is capable of inducing DNA double-strand breaks in intestinal cells, triggering chromosomal instability, gene mutations and cell transformation [217,218]. Increased *E. coli* pks(+) occurs not only in IBD but also in CRC, suggesting a promotional role of this peptide in CRC [182,218,219,220]. The reason for the increased amount of pks(+) *E. coli* in the mucosa observed in some CRC patients is still unclear. Recent studies on the role of tumor-resident *E. coli* in CRC have shown that this bacterium can disrupt the gut vascular barrier and reach the liver via the hematogenous route. These intratumor bacteria boost the formation of a premetastatic niche [221].

A recent metaproteomic analysis showed that among microbial proteins, 341 differed significantly in abundance between CRC patients and healthy volunteers. The variable abundance of microbial proteins related to iron transport, oxidative stress and DNA replication, recombination and repair has been demonstrated as a result of high local iron concentration and high oxidative stress in the large intestine of CRC patients [222]. It has also been shown that certain species of fungi, archaea and viruses can distinguish CRC patients and healthy controls in multiple geographic cohorts. In total, 88 species of bacteria, 108 fungi, 38 archaea and 115 viruses were identified, with varying abundances between people with CRC and healthy individuals. An increase of 48 bacterial species was found in CRC patients, including the widely reported *F. nucleatum*, *P. micra*, *Porphyromonas asaccharolytica*, *Desulfovibrio desulfuricans* and *Akkermansia muciniphila*. However, such species as *Clostridium butyricum*, *Roseburia intestinalis* and *Butyrivibrio fibrisolvens* were reduced in these patients compared to controls. Ecological network co-occurrence analysis revealed associations between bacterial and fungal species such as *Talaromyces islandicus* and *Clostridium saccharobutylicum* in CRC [223].

Interesting research concerns the influence of microbiota-specific T cells on immunity against CRC. In a mouse model of CRC, it was shown that the introduction of *Helicobacter hepaticus* (*H. hepaticus*) increases tumor infiltration by cytotoxic lymphocytes and inhibits tumor growth. Antitumor immunity was dependent on CD4+ T cells (and not CD8+ T cells), B cells and NK cells. *H. hepaticus* colonization induced *H. hepaticus*-specific follicular helper (Tfh) cells, increased the number of colonic Tfh cells and supported the maturation of tumor-adjacent tertiary lymphoid structures. The introduction of immunogenic enteric bacteria may therefore promote Tfh-related antitumor immunity in the colon [10].

Few studies concern the impact of SST/SSAs on the intestinal microflora and/or the phenomenon of bacterial translocation (BT), i.e., the transfer of intestinal microorganisms or their products (endotoxins) from the epithelium to the lamina propria and from there to the mesenteric lymph nodes (MLNs) and then to distant organs [224]. In the study in DSS-induced colitis mice, no significant BT events were observed in either the control or the OCT alone group [202]. In other animal models (rats, pigs), such studies revealed an increased content of bacteria in the cecum and BT in animals receiving OCT (study group) compared to the group without OCT administration (control). Within the study group with BT symptoms, a significant increase in the bacterial content in the cecum was also found. It was accompanied by submucosal edema. OCT appears to significantly increase bacterial counts and BT in rats [225]. Studies in pigs receiving OCT also showed positive bacterial cultures in more than 40% of the animals. Live bacteria were also obtained from MNLs, the liver and the spleen. BT was not observed in control animals. There was a significant difference between both groups comparing BT to MLNs [226]. It has also been observed that SST and OCT are involved in inhibiting the translocation of bacteria such as *Salmonella* from the intestinal lumen into the blood circulation and reducing the bacteria-induced secretion of TNF-α, IL-8 and IL-1β in intestinal epithelial cells [196].

Although SST plays a significant role in GIT function, there is a lack of research on its direct interactions with the gut microbiome. Clinical associations mostly concern patients with acromegaly, diarrhea and individuals on a low-protein diet, not CRC [227]. Regarding intestinal cancers, one study in patients with midgut NETs (*n* = 30) and CD patients (*n* = 50) showed a striking depletion of *F. prausnitzii* in the stool. Microbiota changes in the two remaining NET groups (foregut and hindgut NETs) were atypical and similar to those observed in patients with chronic idiopathic diarrhea. Administration of SSAs did not affect *F. prausnitzii* concentration [228].

Already in the 1990s, the production of SST-like material (SRIF-14 and SRIF-28) was detected in *Bacillus subtilis* and *E. coli* [229,230]. These findings support the suggestion that vertebrate-type NPs have an early evolutionary origin. At a similar time, SST production was also demonstrated by a representative of the fungi kingdom, i.e., *Saccharomyces cerevisiae* (*S. cerevisiae*) [231]. It should be added that *S. cerevisiae* is the main component of the human intestinal microflora and plays a beneficial role in the intestines [232,233]. In a mouse model of DSS-induced colitis, it was shown that *S. cerevisiae* supplementation increased the expression of anti-inflammatory cytokines (IL-4 and IL-10), while reducing the disease activity index and the expression of pro-inflammatory cytokines (TNF-α, IL-6 and IL-17F). Additional effects of this fungus included repair of the intestinal barrier and potential protective mechanisms reducing the level of reactive oxygen species in the colon, inhibiting endoplasmic reticulum stress and regulating intestinal microflora [232]. In adenoma and CRC, a ~3–4 times lower occurrence of *S. cerevisiae* was observed compared to the control group. In vivo studies have shown that *S. cerevisiae* reduces CRC progression mainly by promoting cell apoptosis. Moreover, it modulates the composition of intestinal microorganisms. The mechanism of *S. cerevisiae’s* impact on epithelial cells may involve the NF-κB and mammalian target of rapamycin (mTOR) signaling pathways, which are downregulated [233].

**Figure 4:**
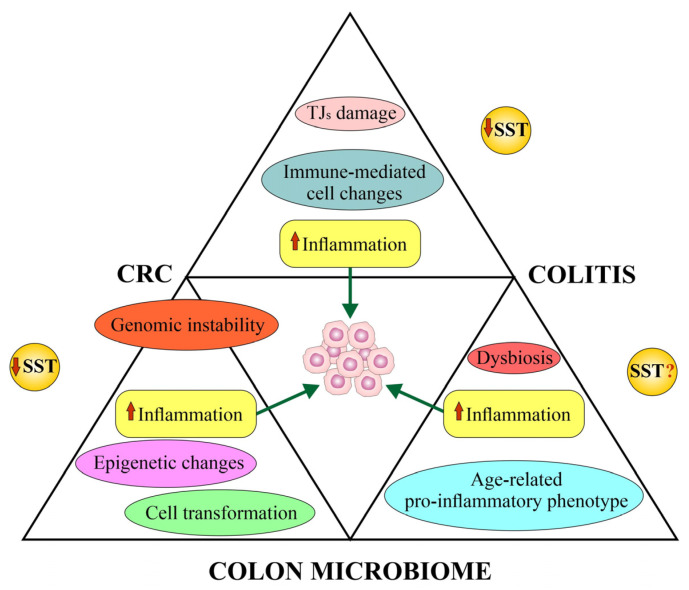
A simplified diagram showing the potential association between colorectal cancer, colitis and colon microbiome. Each side of the “infernal triangle” represents interactions between two states. Increased inflammation is the link between all three conditions. The increased inflammatory process results in immune-mediated morphological cell alterations, damage to the TJs and mechanical disruption of the intestinal barrier, allowing bacteria to translocate into the intestinal lumen. Microbes and their products exacerbate the inflammatory process and can be genotoxic. This results in chromosomal instability, gene mutations and transformation of intestinal epithelial cells. Chronic inflammation leads to dysbiosis and the growth of bacteria (e.g., *E. coli* that produce colibactin), can damage DNA and can stimulate tumor growth. However, the exact relations between colon microbiome, colitis and CRC are still unclear. The reduced secretion of SST in colitis (due to a decrease in SST-producing cells/nerves) and in CRC (due to epigenetic changes in the *SST* gene) results in impaired biological effects of the SST in patients with colorectal cancer. The role of SST in controlling colon microbiome is still not determined. [↓/↑: reduced/increased expression/activity; ?: unclear role; CRC: colorectal cancer; SST: somatostatin; TJs: tight junctions].

Figure 4 represents a simplified summary of the association between colorectal cancer, colitis and the colon microbiome. The potential involvement of SST in these conditions is also highlighted.

## 5. Epigenetic Alterations of *SST* Gene in CRC and Implications for Anticancer Effects

DNA methylation and histone modification are early events in the occurrence and development of GIT cancers, including CRC [234,235,236]. The somatostatin gene is localized on chromosome 3 and has one transcript (splice variant) and 262 orthologues [237]. Epigenetic changes in SST and SSTRs have been described in NETs as well as in sporadic CRC [97,238,239,240,241]. Mori et al. showed SST methylation in 88% of patients with primary colon cancers [238]. A successive increase in SST promoter methylation has also been described from the juvenile colonic epithelium (3.5% ± 1.9%), through the epithelium in healthy adults (~10%) to the developed CRC (30.2% ± 11.6%) [97]. Higher SST methylation was demonstrated in low-level microsatellite stability (MSI-L) than in cancers without MSI-L. SST methylation was accompanied by a reduction in SST mRNA expression [238].

It was also shown that preoperative serum levels of methylated SST were significant prognostic factors for cancer recurrence, in addition to vascular embolism, perineural invasion and CEA level [242]. It was confirmed that SST methylation in pre-operative sera may even be an independent prognostic marker for assessing the risk of CRC and cancer-specific death and recurrence [239].

Studies using bioinformatic techniques have confirmed that increased *SST* methylation results in the downregulated expression status of this gene in the CRC. After constructing a protein–protein interaction network for hypermethylation low expression genes, the five most important genes were identified in this group, including SST [240]. Another study based on differentially methylated regions of the *SST* in CRC by The Cancer Genome Atlas (TCGA) database analysis indicated that the average methylation rate of the *SST* 1stExon was negatively correlated with the *SST* expression in CRC and gastric cancer. The demonstration of this site-specific hypermethylation of *SST* 1st exon allows for the determination of a higher risk of GIT cancers (including CRC) and its use as a potential prognostic marker [243].

In summary, SST epigenetic changes are of great importance in the pathogenesis and clinical presentation of CRC. They contribute to the weakening or lack of antitumor activity of native SST. They can be used as a potential prognostic marker. The possibility of targeting enzymes involved in the epigenetic mechanism to modify the expression of SST and/or SSTRs to improve therapeutic options is discussed [241].

## 6. Somatostatin—Implications for Cancer Therapy

### 6.1. Somatostatin versus Somatostatin Analogues: Structural Characteristics, Pharmacokinetics, Pharmacodynamics and Bioavailability

SST is an endogenous cyclic tetradecapeptide hormone that is secreted mainly by the central nervous system (CNS), GIT, peripheral neurons and pancreatic D cells. It has several biological functions, but it is mainly responsible for inhibiting the secretion of other hormones and neurotransmitters. In both physiological and pathological conditions, endogenous SST also exhibits inhibition of cell growth [15,23,25,89]. The human gene encoding SST is localized on chromosome 3 and has one transcript and represents the ancestral gene of the family. It contains a single intron, which interrupts the coding sequence in the propeptide region of the molecule. The biological activity of the SST protein can be found in both the 14-AA form (SST-14) and the 28-AA form (SST-28), which both come from the larger precursor preprosomatostatin [57,92,237].

First isolation and characterization of SST-14 as a tetradecapeptide with a sequence of H-Ala-Gly-Cys-Lys-Asn-Phe-Phe-Trp-Lys-Thr-Phe-Trh-Ser-Cys-OH was performed on material derived from sheep hypothalamus [14]. The cyclic structure of the native SST molecule was discovered to be linked by two intramolecular disulphide bonds between the two cysteine residues. The same biological activity was demonstrated for the reduced linear and cyclic forms of SST-14 in vitro, so the cyclic form was not necessary for recognition by specific SSTRs [244]. SST-14 is chosen as a therapeutic peptide because of its universal high nanomolar attraction to all five receptor subtypes (SST1-5) [137]. SST-28 is a second native form of SST, reported for the first time in 1980 [245]. This molecule contains the AA sequence of SST-14, extended by 14 residues at the N-terminus. Both SST-14 and SST-28 have a cyclic structure and derive from single precursor. While the distribution of two active forms of SST is similar, SST-14 is the predominant form in the CNS, in the enteric neurons and in most peripheral organs, including GIT. In contrast, SST-28 is mainly produced by intestinal mucosal EECs [57,191,246].

Enhancers and silencers in the gene promoter region, as well as the binding of transcription factors to these elements, play a major role in the mechanisms of regulation of SST secretion. The role of pre-translational mechanisms regulating the expression of this peptide (e.g., methylations and polymorphisms in the gene promoter region, activity of various transcription factors) and post-translational mechanisms (e.g., proteolytic cleavage of preprosomatostatin to SST-14 and SST-28 and secretion of the peptide) was also confirmed [97].

SST is secreted mostly in a paracrine/neurocrine fashion, released in a pulse manner as a very short-lived peptide of about 3 min bioactive half-life in circulation, where it is degraded rapidly by ubiquitous peptidases [22,43,57]. Following intravenous (i.v.) administration of ^3^H-labeled SST, the total body clearance was ~50 mL/min. In man, the value was calculated to be as high as 3000 mL/min, which greatly exceeds the hepatic blood flow [247]. The actions of SST as an endogenous SSTR ligand are mediated via signaling pathways of type G protein-coupled receptors. SST-14 binds with higher affinity to SST1-4, whereas SST-28 mainly interacts with SST5 [15,19,20,21,57,90].

The clinical use of native SST is limited by its very short half-life and the broad spectrum of biological responses [22,43,57]. As a result, SST receptor-selective agonists/SSAs have been created. Many strategies are used to increase the stability of peptides for therapeutic purposes. Natural AA sequences are optimized by introducing conformational constraints (i.e., caused by cyclization or insertion of unnatural AA into peptide sequences), ensuring unfavorable changes in binding entropy and conjugation with glycosylated molecules or polyester compounds at the N-terminus of synthetic peptides. Other activities include the formation of dimers, tetramers or heterodimers, which improve the stability and affinity of synthetic peptide chains for their receptors. Among the few known cyclized peptides include precisely SST (reviewed in [248]). Most SSAs contain a disulphide bond and peptide chain shorter than the parent sequence [249].

Compared to endogenous SST, SSAs have several advantages. They have been made to maintain their therapeutic effect for a longer period, which results in less frequent dosing and increased patient comfort. SSAs show greater selectivity and are more biologically stable [22,25,28,43,57,89]. In addition to better efficacy and better therapeutic index, SSAs are free of serious side effects [23,89].

Three of them have been approved in clinical practice to date, with lanreotide (LAN) and octreotide (OCT) being first-generation SSAs and pasireotide (PAS) being a second-generation SSA [22]. The majority of SSAs bind strongly to two of the five receptor subtypes [43]. Moreover, two subfamilies of SSTRs have been described on the basis of chemical structure identity and pharmacological characteristics: the first class, comprising SST2, SST3 and SST5, binds OCT and LAN, while the second-class receptors, SST1 and SST4, do not interact with these SSAs [250].

Among SSAs, octreotide (OCT) is the most extensively studied [22,25,28,43,57,89,249]. It was the first biostable SSA with a strong affinity for SST2 and SST5. OCT is a synthetic peptide analogue with eight AA that was engineered to overcome the limitations of native SST [244]. This SSA with code-named SMS 201-995 has a longer half-life (90 min) compared to native SST (~1–3 min) and the duration of activity after subcutaneous (s.c.) administration lasts ~8 h. It is three times more potent in vitro and up to 70 times more active in vivo than native SST [249]. Increased biological activity and reduced metabolic degradation were achieved by the insertion of the D-Phe at the N-terminus and the amino alcohol Thr-ol at the C-terminus [248]. Both native SST and OCT have a high affinity for specific receptors and IC50s in the subnanomolar range. In animals and humans, investigations of the pharmacodynamics/pharmacokinetics relationship between SST and OCT show plasma levels of 0.2–0.5 ng/mL (approximately 0.3 nM) to be therapeutically relevant for both of them. Following i.v. administration of ^14^C-labeled OCT, the total body clearance was ~4.2 mL/min [247]. The pharmacodynamic features of OCT are similar to those of SST, with a wide spectrum of inhibitory effects on anterior pituitary function, pancreas and gut endocrine secretions and other GIT functions. Compared with SST, OCT is highly resistant to enzymatic degradation. In blood, OCT is mainly distributed in the plasma, with 65% bound to lipoproteins. The s.c. injection appears to have rapid and complete absorption, with bioavailability estimated at 100%. Mean peak plasma concentrations are between 2 and 4 μg/L in patients receiving 50 to 100 μg. Peak concentrations are reached within 20 to 30 min and are 20 to 40% of corresponding values after i.v. injection [251]. OCT is used s.c. or i.v. in several daily injections. The bioavailability is similar in both routes of administration. The serum concentration of OCT increases linearly with increasing doses, regardless of whether the drug is given s.c. or i.v. The average bioavailability of an s.c. dose is 100% or greater. Distribution is rapid. The elimination half-life of OCT by either route of administration is ~1–5 h (30 times longer than that of SST). About 32% of the s.c. dose is excreted in the urine as unchanged OCT. It is worth noting that OCT retains some activity on oral administration [244]. In addition, it should be emphasized that OCT (SMS 201-995) marketed as Sandostatin^®^ (50, 100 and 200 μg s.c. every 8–12 h) is the first SSA to be approved by the Food and Drug Administration (FDA). It is available in both conventional and modified long-acting release (LAR) injection (Sandostatin LAR^®^). The Sandostatin LAR^®^ formulation contains OCT distributed within polymer microspheres and it is available for intramuscular injection at doses of 10, 20 or 30 mg every 28 days (reviewed in [22]).

Another available SSA is lanreotide (LAN, Somatuline), which has similar effects to OCT. It was developed in the 1990s with the intent of developing a longer-acting SSA. BIM-23014, one of its initial formulations, had a half-life of 90 min. A slow-release formulation, LAN sustained release (LAN SR), followed soon after and has a half-life of 4.5 days. It was designed as microspheres made of biodegradable polymers and is available for intramuscular injection at doses of 30 or 60 mg every 7–14 days. LAN Autogel^®^ (ATG), a sustained release aqueous formulation, was presented as a prefilled injection for s.c. administration at doses of 60, 90 or 120 mg every 28 days [22,23,252]. OCT and LAN represent the first generation SST receptor ligands (SRLs) [25].

Pasireotide (PAS, SOM230), a novel SSA, represents a second-generation SRL. It binds with higher affinity to SST1 (30-fold), SST3 (5-fold) and SST5 (39-fold) and with the same affinity to SST2 (three-fold) when compared with OCT and with higher affinity to SST1 (19-fold), SST3 (nine-fold) and SST5 (106-fold), but with the same affinity to SST2 (two-fold) when compared with LAN [253]. Pasireotide LAR (Signifor LAR^®^) was approved in 2014 by the FDA and it is available for intramuscular injection at doses of 20, 40 or 60 mg every 28 days [22].

It was believed that OCT, RC-160 (Vapreotide) and LAN (BIM-23014) were the most active, but attempts are still being made to develop more perfect forms of these drugs [23,109,118,120,136,158,159]. Recently (2022), an oncolytic virus delivery system was also introduced to express the SST fusion protein, which was expected to result in the combined antitumor effects of both SST and oncolytic virus to destroy tumor tissues [137].

In the context of the present review, most of the SSAs have been used in both in vivo and in vitro experiments to demonstrate the antitumor effects of the ligand-receptor system in CRC (Table 1 and Table 2).

### 6.2. Somatostatin Analogues in the Therapy of Colon Neuroendocrine Tumors

The antiproliferative and pro-apoptotic activities of somatostatin analogues (SSAs) are mainly used in the treatment of highly differentiated GEP-NENs and other tumors that express SSTRs [22,46,183,254,255,256,257]. However, treatment in SSTR-negative NETs are also presented [254]. Class I evidence for the antiproliferative and antitumor effects of SST was provided by the CLARINET clinical trial in patients with GEP-NETs with grade 1 or 2 disease (Ki-67 < 10). It showed that LAN Autogel improved progression-free survival (PFS) [258,259]. The promising outcomes of this therapy as a maintenance treatment following the first-line treatment in aggressive G1/2 duodeno-pancreatic NETs require confirmation [260].

The somatostatin analogue OCT acetate was initially introduced in 1987 and in the form of LA in 1998 for the treatment of carcinoid syndrome, given its ability to inhibit hormone secretion by NETs [87]. An interesting design of a new peptide composed of four synthetic NP analogues (VIP, bombesin, SP and SST) with code-name DRF 7295 was presented [158,159]. Both in vitro and in vivo studies (mice, rabbits) have shown that GIT tumors (including CRC) are highly sensitive to this combined peptide [158]. In the mechanisms of action of this SSA, the authors emphasize a ~58% reduction in cAMP levels and EGF-dependent phosphorylation of MAPK (ERK1/2), an increase in p53 levels, downregulation of Bcl-2 levels in Colo205 cells, inhibition of VEGF secretion and induction of active caspase-3 in HT-29 cells. Moreover, inhibition of capillary tube-like formation in ECs has been demonstrated [159].

Based on many preclinical studies, it was determined that first-generation SRLs (e.g., OCT and LAN) prefer SST2 in binding. However, second-generation SRLs (e.g., PAS) have a high affinity for many SSTRs (mainly SST5, SST2 and SST3). These analogues differ not only in their affinity for SSTRs but also in other biological effects [25]. A recent review on LA OCT, LAN and PAS therapy in patients with NETs (including colon and rectum NETs) showed that LA SSAs are an effective and safe initial therapy in patients with well-differentiated NETs, enabling long-term control of tumor growth and clinical symptoms [257]. Currently, OCT LAR and LAN Autogel are the cornerstones of systemic therapy for NETs, either alone or in combination with other targeted therapies, as confirmed by the literature data [25].

Taking into account the study on colon NETs, stable disease (SD) was demonstrated in 57% of patients [261] or in nearly 80% of rectum NET patients after OCT therapy [262]. A summary of the current preclinical and clinical data concerning the treatment of NENs with SST–dopamine chimeric molecules presents one of the latest excellent reviews [263]. Heterodimerization between these receptors is associated with increased anticancer activity. This evidence has contributed to the development of research on new chimeric multi-target molecules. A new chimeric compound called TBR-065 (formerly BIM-23B065) targeting SST2, SST5 and the dopamine 2 receptor (D2R) appears to be a novel molecule with significant prospects in the therapy of well-differentiated NENs, as demonstrated in several preclinical studies and preliminary clinical trials [263]. Recently, another second-generation, ligand-based chimeric antigen receptor (CAR) was developed containing OCT in its extracellular molecule. It was demonstrated that anti-SSTR CAR T cells exerted antitumor activity against SSTR+ NET cell lines in vitro and in vivo. Therefore, it is a potential candidate for early phase clinical trials in patients with NETs [264].

### 6.3. Somatostatin Analogues in the Therapy of Colorectal Cancer (CRC)

CRC is listed among non-endocrinological tumors for potential treatment with SSAs, but the therapeutic effects are still uncertain [22,42]. The presence of SST receptors on cells is considered a predictive factor for the effectiveness of SSA therapy. In sporadic CRC, results in the expression of SSTRs vary widely. Studies on the expression of SSTRs in pure adenocarcinoma are characterized by high heterogeneity of the obtained results. However, it seems that the most frequently represented subtypes of SSTRs in this tumor are SST2 and SST5. The greatest correlation with clinical data was demonstrated in the case of SST2, followed by SST5. As for the prognostic value of receptor expression, the results are surprisingly divergent and also concern mainly SST2 and SST5 (reviewed in [92]). However, the occurrence of SSTRs does not determine their functional status. It is important to remember the presence of various receptors within tumor cells and the heterodimerization of SSTRs, which may change their functional response. SSTRs can also dimerize with other GPCR members, e.g., D2R, or with other receptor families, e.g., tyrosine kinase receptors (EGFRs) [20,21,263,265].

Attempts have been made to use SSAs in non-endocrine CRC [101,102,104,266,267,268]. The use of various SRLs ranged from the lack of a spectacular therapeutic effect in patients with CRC (*n* = 4) [266,269] to the presence of SD in a certain group of patients (4/16) or a periodic improvement in the quality of life with a reduction in pain in most of the respondents [104]. One of the first forms of clinical evidence of the inhibition of RC growth (*n* = 12) by an SST analogue (SMS 201.995.) was presented in the early 1990s. After treatment with this SSA, a decrease in serum CEA level was observed in 2/4 of patients with previously elevated levels of this marker and a decrease in tumor proliferative activity (Ki-67 expression) [101].

A phase III clinical trial administering OCT (150 μg s.c. three times daily) in patients with advanced asymptomatic CRC (*n* = 260) showed comparable mean time to tumor progression and mean survival versus control group (~17 months). The poor effects of OCT therapy were probably due to the loss of SSTR expression in patients with lymph node metastases or distant metastases. This suggests the possible effectiveness of SST only in a limited number of patients with tumors expressing specific subtypes of SSTRs [266]. In turn, administration of OCT (200 μg three times a day, 5 days a week) to patients with GIT tumors resistant to chemotherapy (including 24 patients with CRC) showed a significant advantage in terms of survival time (36 weeks) versus the control group (24 weeks) who received supportive care. Additionally, 11/24 patients receiving OCT showed SD compared with only three in the control group. OCT therapy therefore appears to provide a survival benefit in patients with advanced chemotherapy-resistant CRC, but these results should be treated with caution. The need for additional studies is indicated to confirm these favorable data and clarify other important issues, e.g., the relationship between SSTR status and response to treatment and between the optimal dose and duration of OCT administration and the impact of OCT treatment not only on survival but also on the quality of life of patients [267].

Although none of the clinical studies have demonstrated a significant impact on the complete regression of the tumor after SSA treatment, the reduction in the proliferative potential, stabilization of the disease, prolongation of patient survival and improvement of the quality of life are important reasons for continuing such studies. The multidirectional effect of SSAs on the GIT (e.g., reducing the secretion of hormones and biologically active substances) and their antiproliferative effect should be used to reduce tumor mass, delay disease progression and extend life [45,92].

### 6.4. Somatostatin Analogues in the Therapy of Selected Non-Endocrine Cancers beyond CRC

SSAs, which have been clinically used to treat NETs for nearly 30 years, have a safety profile that benefits patients with non-neuroendocrine tumors other than CRC [22,23,25,28,42,43,89]. They are increasingly used in the treatment of human cancers such as breast cancer (BC), prostate cancer (PC), lung cancer (LC) and hepatocellular carcinoma (HCC), especially those with the confirmed presence of SSTRs. SSAs are used as monotherapy or in combination with other forms of therapy. However, treatment effects are highly variable, and the antitumor role of SSTs in these cancers is still unclear [22,270].

#### 6.4.1. Breast Cancer (BC)

BC in women is the most frequently diagnosed malignancy, which has even overtaken lung cancer [2]. In this cancer, tumor growth inhibition and/or antiproliferative effects of SSAs have been studied in vitro and in animal models [109,118,120,121,136,137]. The most commonly used SSA was OCT [271,272] or its modifications [109,118,120,121,139].

Already in the 1990s, direct inhibition of the growth of BC cells (MCF-7) by the natural hormone SST-14, as well as Sandostatin, and another SSA–CGP 15-425 was demonstrated [271]. Keri et al. showed that TT-232 was effective in inhibiting the growth (from ~44% to nearly 90% inhibition) of four types of BC cell lines, as well as in the case of MDA-MB-231 human BC xenografted in mice. The mechanism of action of TT-232 showed a reduction in the content of PTP in human EGF receptor 3 (HER3)-positive BC cells (MDA-MB-453) [121,139]. On MCF-7 cells, it was shown that the action of OCT (SMS 201-995) leads to apoptosis, which was associated with a rapid, time-dependent induction of wt p53 and an increase in Bax [272]. Studies on MDA-MD-23 cells using various SSAs [109,118,120,137] showed variable results. Thus, for example, the compound 2 (D-Phe-c(Cys-Phe-D-Trp-Dap-Tle-Cys)-Thr-NH2) had antiproliferative effects on MDA-MB-231 cells with the IC50 0.03 mM [118]. Even better antiproliferative effects were obtained after using conjugated CTX-OCT loaded onto Ca–alginate beads (CTX-OCT-Alg) compared to free drug in MCF-7 cells [136]. Recent research by Fan et. al. demonstrates the use of the oncolytic virus delivery system to produce the VG9/(SST-14)2-HAS vaccine, which is characterized by a complex antitumor effect on SSTR-positive tumor cells in vitro. However, as the study showed, none of the SSTRs were expressed in MDA-MB-231 cells. To verify the oncolytic potency of VG9/(SST-14) 2-HSA on different tumor cells (including MDA-MB-231), it was shown that these cells were sensitive to all three viruses, and no significant difference was observed in the high oncolytic activity of the viruses [137].

Other attempts to use SSAs in the treatment of BC include combining OCT with other drugs, e.g., with Paclitaxel (taxol) [273], Daunorubicin plus dihydroartemisinin liposomes in MDA-MB-435S cells and xenografts [274] or SSTR targeted liposomes in combination with Diacerein (DN) [275]. The OCT-conjugate taxol retains the biological activity of taxol in inducing the formation of tubulin boundles, ultimately causing apoptosis of MCF-7 cells [273]. The liposomes displayed a prolonged circulating time in vivo, more accumulation in tumor location and a robust overall antitumor efficacy with no evident toxicity at the test dose in MDA-MB-435S xenograft mice [274]. Enhanced apoptosis in BC cells was detected in SST–Diacerein-loaded liposome (DNL)-treated groups and more effectively inhibited the oncogenic IL-6/IL-6R/STAT3/MAPK/AKT signaling pathways as compared to DN or DNL in cancer cells [275].

An interesting therapeutic concept in BC is also to exploit the presence of EGFR subtypes (ErbB1-4) in coexpression with SSTRs (reviewed in [23]). It was confirmed that BC tumor tissues express all five SSTRs and four EGFRs [276]. The mechanisms of SST3 and antiproliferative and pro-apoptotic actions have been shown in MCF-7 and MDA-MD-231 cells [277].

Clinically used Somatuline (LAN) therapy in combination with Tamoxifen (TMX, TAM) in postmenopausal untreated BC patients gave beneficial effects. A reduction in serum IGF-1 concentration (without changes in GH level) was observed. Approximately 12% of patients exhibited a complete response and 37.5% a partial response for an overall response rate of 50% (95% CL 35–69%) [278]. The use of another SSA, i.e., RC-160 (Octastatin, Vapreotide), in women with previously treated metastatic BC also reduced IGF-1 levels [279]. Good tolerance of SSAs was generally observed [278,279], and only an increase in fasting blood glucose level was observed [279]. However, other pilot studies using Lanreotide (BIM-23014) did not confirm the effectiveness of this SSA in advanced BC therapy [280]. In the NCIC Clinical Trials Group MA.14 in an early stage BC study, postmenopausal women used TMX or TMX-OCT (90 mg as monthly intramuscular depot injections) as adjuvant therapy. Although OCT-related changes in circulating IGF-1 and C-peptide levels were significantly reduced, this treatment did not provide significant clinical benefit [281].

#### 6.4.2. Prostate Cancer (PC)

PC is the most common tumor in older men [2]. The presence of SSTRs (mainly SST2, -3 and -4) is also demonstrated by normal and cancer cells of the prostate gland [23]. Both in vitro and in vivo studies have also shown that SSAs exert a significant inhibitory effect in various prostate cell lines and models of PC [282,283,284]. In an animal model (syngeneic Dunning R-3327-H prostate tumors in male rats), Somatuline (BIM-23014C) was administered as a therapeutic agent. Treatment of tumors in castrated animals with this OCT produced a significant tumor suppressive effect that was greater than that produced by castration alone. The inhibitory effect on PC growth was also inducible in tumor-bearing animals that had already escaped castration inhibition. The relative nontoxicity of Somatuline suggests that chronic or maintenance therapy for slow-growing PC may be used in the clinical setting [285]. At an early stage of tumor development, the growth of androgen-independent PC can be suppressed by the use of RC-160 and the bombesin/gastrin-releasing peptide (GRP) antagonist (RC-3095) [282].

In the rat PC model, attempts have been made to improve the clinical response in advanced cancer by combination therapies, e.g., by administering SSA with D-Trp6 luteinizing hormone-releasing hormone (D-Trp-6-LH-RH) microcapsules. The inhibition of tumor growth was greater than that caused by RC-121 alone [286]. Comparative analysis of histopathological changes after treatment with various SSAs (RC-121, RC-160) and D-Trp-6-LH-RH showed a significant reduction in tumor weight in all treated groups. The greatest decrease in tumor volume was observed in the groups receiving the combination of SSA and D-Trp-6-LH-RH [287]. Studies on the role of AN-238 showed a simultaneous growth suppression and a significant increase in apoptosis in nude mice bearing s.c. xenografts of PC-3 human androgen-independent PC cells [284]. As shown by in vitro studies conducted by Brevini et al., SST-14 had a direct inhibitory effect on PC cell (LNCaP) proliferation and protein secretion, two effects probably mediated by the activation of PTPs [283].

The clinical use of OCT (SMA 201-995) in the treatment of patients with advanced hormone-refractory PC did not produce spectacular results. Six of twenty-four patients received salvage chemotherapy after the disease progressed on SMS 201-995 therapy, five of whom had achieved objective tumor regressions. The authors conclude that the use of OCT even increases the growth of PC, but the use of this SSA may sensitize PC to chemotherapy [288]. In turn, in the group with a similar PC using LAN (BIM-32014), improvements in performance status (40%) and bone pain (35%) and decreases in prostate-specific antigen (PSA) levels (50%) and SD (16%) were demonstrated. The 1-year global survival rate was 72% [289]. Other authors have conducted a dose-escalation study of LAN (Somatuline) in 25 patients with hormone-resistant, metastatic PC, without acquiring a clinical response based on radiographic criteria or tumor markers [290].

Previously published studies have documented clinical response in many treated patients with significant improvement in parameters related to quality of life. In light of these promising results, large-scale randomized controlled trials are warranted to precisely determine the role of LAN and other SSAs in the treatment of patients with castration-resistant-stage (CRPC) PC [291].

#### 6.4.3. Lung Cancer (LC)

LC is the second most common cancer worldwide and remains the leading cause of cancer deaths [2]. SSTRs may be expressed particularly by small-cell lung cancer (SCLC) and bronchial carcinoid disease [292,293,294]. In the case of bronchial carcinoid and acromegaly, the clinical effectiveness of OCT (50 μg) was observed, while in vitro studies showed the inhibitory effect of both OCT and SOM230 (Pasireotide) on the reduction in GH and GHRH secretion [293].

The presence of SSTRs was confirmed in 3/4 of established SCLC cell lines but not in two non-SCLC cell lines. OCT (SMS 201-995, Sandostatin, 10^−9^ M) inhibited the growth of one of three SSTR+ SCLC cell lines ((HX149 cells). None of the SSTR-negative cell lines were inhibited by OCT. Interestingly, no inhibitory effect of OCT was observed in NCI-H69 cells, which showed high levels of SSTRs [295]. Other authors have shown that SCLC has higher expression of SST2/SST5 but lower SST3 and SST1 compared to lung adenocarcinoma or squamous cell carcinoma [296].

Pioneering trials of the clinical use of OCT (250 μg three times a day) in 20 patients with SCLC (both before and after chemotherapy) did not give promising results. A reduction in serum IGF-1 was observed to 62 ± 7% of pre-treatment levels. However, there was no evidence of anti-tumor activity as measured by tumor weight or serum neuron-specific enolase (NSE) levels [295]. In another study, after the initial demonstration of the presence of SSTRs, LAN (30 mg Somatuline^®^) was administered to 54 patients with SCLC with limited disease (LD). A better average survival time and a longer time to disease recurrence were observed in patients treated with OCT combined with chemotherapy (Paclitaxel + Carboplatin) compared to patients treated with chemotherapy alone [297].

A large, multicenter, randomized phase 3 study (G04.2011 trial) conducted in SCLC patients expressing SSTRs and using LAN as a maintenance treatment after response to standard treatment did not show a significantly longer survival in SCLC. Only a modest PFS benefit was observed in LD SCLC. These results deserve further research [298].

#### 6.4.4. Hepatocellular Carcinoma (HCC)

HCC is the most common primary liver cancer and better treatments are still being sought due to high recurrence rates (>50%), even after aggressive therapies [270]. Differential expression of SSTRs was confirmed in selected HCC cell lines (e.g., SMMC-7721, HepG2 and BEL-7402), normal liver cells (L-02) [299] and in HCC tissues in vivo [300,301]. Numerous studies demonstrate in vitro antiproliferative and pro-apoptotic effects of both OCT [299,302,303] and LAN [304]. Some authors observed only minor changes in cell proliferation and morphology after short-term OCT therapy on HCC cells (BEL-7402), with no effect on apoptosis. However, long-term OCT treatment effectively inhibited the development and growth of HCC, probably through resensitization and upregulation of SST2 [302].

Most clinical trials in HCC used LA OCT (Sandostatin). Several randomized trials with this type of SSA have been performed, lasting from several months to 5 years. Very different results were obtained [270]. Examples of randomized trials show both an extension of the average survival time of patients after using OCT and a decrease in AFP concentration [301,305], as well as a lack of impact of this SSA on life extension, tumor regression or changes in AFP concentration in advanced HCC [300].

The randomized double-blind HECTOR trial also failed to demonstrate a survival benefit in HCC patients treated with Sandostatin LAR (30 mg, intramuscularly every 4 weeks) compared to placebo [306]. Another phase III multicentre, randomized study in patients with advanced HCC confirmed that this form of OCT at the given dose has a favorable safety profile, and 33% of patients achieved stabilization of the disease for a mean time of 5.5 months (95% CI, 1.1–9.9). However, this therapy did not improve OS and may have had a negative impact on quality of life [307]. Contrarily, in another study lasting 3 years, a significantly longer survival time was observed in the OCT group (49 ± 6 weeks) compared to the control group (28 ± 1 week) and the SSTR negative group (28 ± 2 weeks) [301].

OCT was also used in combination with TMX [308,309] and nonsteroidal anti-inflammatory drugs: rofecoxib [310] and celecoxib [311]. TMX + OCT combination therapy in patients with unresectable HCC was superior to the effect of 5-FU and mitomycin C in terms of increasing survival rate, prolonging survival time and reducing side effects [308]. However, Verset et al. did not prove any differences in terms of reducing AFP levels, tumor regression, improving quality of life and preventing variceal bleeding between both groups. The OCT + TMX combination did not affect survival, tumor progression and quality of life in patients with advanced HCC [309]. In combined treatment with OCT + rofecoxib, mean OS (154 days) and mean time to tumor progression (94 days) were also not different for both treatments [310]. However, the use of transarterial chemoembolization (TACE) with celecoxib and LAM prolonged OS, increased tumor response, reduced post-embolization syndrome and was well tolerated by patients with unresectable HCC [311]. The role of SSAs based on in vitro and in vivo studies, as well as the use of various SSA preparations in patients with HCC, is described in detail in the latest review [270].

## 7. Concluding Remarks and Future Directions

The antitumor activity of SST and its analogues in CRC includes direct and indirect actions. SST epigenetic changes (mainly methylations) are observed in a varying percentage of CRC (30–88%). This may result in a reduction in the local expression of SST (mRNA, protein) and dysregulation of the antitumor function of the endogenous peptide in these patients.

In studies of the antitumor activity of SST in vivo and in vitro, most attention has been paid to its antiproliferative and pro-apoptotic effects. In vivo studies indicate the potential impact of SST on regulating the expression of cyclins (e.g., D1, A, E) and cyclin-dependent kinases (e.g., CDK2, CDK4), which results in blocking the cell cycle. An inverse correlation was demonstrated between the effects of SSAs and tissue expression of proliferation markers (Ki-67 and PCNA) in the CRC. The pro-apoptotic activity of SST is supported by the results of in vivo studies, which showed an increase in the expression of apoptosis proteins (Bax, Fas, caspase 3 and 8) and a decrease in the expression of anti-apoptotic proteins (Bcl-2) in correlation with the expression of SST. The effect of SST on p53 expression in CRC is more diverse, although an increase in the expression of this protein has been reported.

The proven antiproliferative and pro-apoptotic mechanisms of SST/SSAs in the CRC include inhibition of cytosolic tyrosine kinase activity and an increase in the activity of phosphatases, mainly PTPs. The signaling pathways involved in the antitumor function of SST are primarily MAPK/ERK/AKT and Wnt/β–catenin. Inhibition of MAPK signaling by SST (via SST3/5) is expected to occur through activation of PTPs and weakening of COX-2 expression in CRC cells. Selective inhibition of human telomerase activity, participation in the translocation of Ku86 from the cytoplasm to the cell nucleus, regulation of the Ku70/86 heterodimer and interaction between Ku70, nCLU and Bax may also contribute to modulating apoptosis and blocking the growth of CRC cells and via the SST system.

The antiangiogenic role of SST/SSAs in CRC is also being studied. However, little is known about the exact mechanisms of this action in vivo. In vitro studies and in animal models of CRC indicate the involvement of SST in reducing the number of tumor blood vessels in connection with antiproliferative and pro-apoptotic effects and inhibition of VEGF expression. Preparations composed of SST and other NPs have a stronger antiangiogenic effect.

The mechanisms of SST action in regulating the immune status in sporadic CRC are the least known. This cancer is closely associated with chronic inflammation; hence, the role of SST has been studied mainly in various models of IBD. Most authors have pointed to the immunomodulatory (mainly inhibitory) role of SST in CRC mucosa and the protective effect on the intestinal barrier. The role of positive feedback between SST and the secretory activity of immune cells in IBD is emphasized. However, due to divergent research results in IBD models, unclear mechanisms of action of SST, differences between colitis models and IBD in humans and different protein expression profiles in lamina propria in IBD and CRC, this study requires continuation. Due to the spectacular quantitative and qualitative composition of microorganisms in the human large intestine, it is important to determine the interaction of the tumor genome/TME/host’s gut microbiome at an increasingly better scale. The role of SST in these associations remains unclear.

The results of SSA treatment for sporadic CRC and other non-endocrine neoplasms (e.g., breast, prostate, lung and hepatocellular carcinoma) in monotherapy are useful in treatment, but the effects are highly variable. The antitumor role of SSTs in these cancers is still unclear.

In the case of CRC, the results of SSAs in monotherapy are not spectacular. An inhibition of tumor growth or its remission has not been proven, although a reduction in the proliferative potential and clinical stabilization of the disease, prolongation of survival and improvement in the quality of life in CRC patients have been observed. An individual approach to therapy using SSAs (personalized therapy) should be considered. Clinical trials on new forms, primarily complex antitumor drugs, with an affinity for a larger number of SSTRs should be continued.

## Figures and Tables

**Table 1:** The antitumor activity of SST in various CRC cell lines and/or animal models using SSAs.

Cell Line/Animal Model	SSA Type/Methods	Findings	Antitumor Activity	Ref. No.
CX1, X56 and HT-29; nude mice xenografts (CX1 and X56 cells)	SST-14	(i) Ʇ tumor growth in CX1 in vivo and HT-29 in vitro (but not X56 cells); (ii) Ʇ the gastrin-induced growth	↓ Cell proliferation Ʇ Tumor growth	[113]
DHD/K12 rat colon cancer cells and implanted tumors	SST-14 and RC-160	(i) Ʇ tumor growth in vivo; (ii) ↓ LI by 35%; (iii) ↓ total protein/DNA in the tumors by 70.1%/68.7%, respectively		[126]
LIM 1215, LIM 1863, LIM 2405, LIM 2412; LIM 2412 and LIM 2405; xenografts in nude mice	SMS 201-995	Ʇ both in vitro and in vivo growth of colon cancer		[122]
LIM 2412 and LoVo; LIM 2412 xenografts	SMS 201-995	(i) a 13% ↓ in CEA (LoVo); (ii) a direct correlation between the mean volume of the xenografts and serum CEA level; (iii) Ʇ xenograft growth correlated with a ↓ serum CEA		[148]
HT-29 and IEC-6	SST-14	Ʇ HT 29 cell growth (only in the presence of serum)		[114]
HT-29, SW620 and other cell line (MCF-7)	TT-232	(i) strong tyrosine kinase inhibitory effect; (ii) Ʇ proliferation (~70%) in both CRC cell lines		[139]
HT-29 and nude mice bearing xenografts	RC-160	(i) ↓ tumor growth; (ii) specific binding sites of SST, bombesin and EGF on intact HT-29 cells or on HT-29 xenografts		[115]
SW480 and SW620	SMS 201-995	(i) Ʇ cell proliferation vs. untreated cultures; (ii) after OCT 10^−8^ M: ↓ the mitogenic effect of EGF on SW480 vs. cells exposed to EGF alone; (iii) ↑ the effect on cell growth by its combination with cytokines (IL-2 and IFN-γ) in SW620		[123]
C170 and LIM 1215	SMS 201-995 (Sandostatin) alone and in combination with 5-FU	(i) SST alone with minimal inhibitory effects on cell growth; (ii) after 5-FU alone: Ʇ as low as 39.6% of C; (iii) after 5-FU + SST: a 10–30% Ʇ vs. 5-FU alone		[127]
SW620	TT-232	(i) a strong Ʇ of cell proliferation; (ii) a rapid and sustained ↑ PTP (5–30 min)		[124]
HT-29, SW620, Colo205 and many other cell lines; transplanted Colon 26 tumor	TT-232	(i) Ʇ cell proliferation >50% in Colo205; (ii) Ʇ tumor growth (70%); (iii) ↑ AI in HT-29 (7×); (iv) Ʇ tyrosine kinase (75%) in SW620 which correlated with the antiproliferative and pro-apoptotic effect		[121]
HT-29	Sandostatin and TT-232	(i) after TT-232: ↓ 59 ± 6% in cell no., after Sandostatin: ↓ 21 ± 12%; (ii) ↓ p86 Ku in cytoplasm at the first 4 h, and ↑ in the nucleus at 1 h followed by a ↓ at 4 h		[116]
HCT-116 and LoVo expressing wtp53; HCT-15 and HT-29 with mp53; nude mice model	AN-238, consisting of 2-pyrrolinodoxorubicin (AN-201) linked to octapeptide SST carrier RC-121; DOX treatment	(i) functional SSTRs on HCT-116, HCT-15 and HT-29; (ii) ↑ p53 activity; (iii) AN-238, AN-201 and DOX equally effective on HCT-116 tumors; (iv) after AN-238: Ʇ growth of HCT-15 and HT-29 cancers		[130]
Caco-2	SST	(i) Ʇ cell growth and modulation of the activation of Ku70/86 heterodimer and the Ku86 levels in the nucleus by ↑ its mRNA level		[145]
C-26 and HT-29 in xenografted mice	TT-232	in xenografts max tumor Ʇ—27%; in C-26 −75%		[117]
Caco-2, HT-29 and HCT-116	SST-14; colorimetric assay; BrDU assay; EIA for PGE2; COX-2 mRNA silencing; RT-PCR, WB for SSTRs, COX isoforms, p-ERK-1/ERK-2 and p-AKT	(i) (+) COX-2 and SST3/4/5 in HT-29 and Cox-2 and SST3/5 in Caco-2; (+) SST2/3/5 in HCT-116; (ii) Ʇ basal COX-2, ꞱPGE2, ꞱDNA synthesis and growth in Caco-2 and HT-29 via SST3 or SST5; (iii) ↓ phosphorylation status of ERK-1/ERK-2 in Caco-2; (iv) ↓ constitutive COX-2 via SST-mediated activation of PTP leading to ꞱMAPK pathway		[134]
HT-29 and other cell lines (MDA-MB-23, HepG2, HeLa, Lep-3)	SMS 201-995 and other modified analogues	(i) all compounds with different concentration-dependent antiproliferative effects against all cell lines except Lep-3 after 24 h		[118]
Caco-2 and HT-29	OCT with or without the trophic effect of insulin treatment; MTT; TRAP; IHC; RT-PCR	(i) (+) SST1/2A/2B/3/4/5 in both lines; (ii) ↓ proliferation of both lines in a time and dose-dependent manner; (iii) ↓ telomerase activity in serum-free cultured medium and ↑ telomerase in the presence of 10% FBS (Caco-2); ↑ telomerase in both conditions in HT-29		[119]
HT-29 and other cell lines (MDA-MB-23, HepG2, HeLa, Lep-3)	Several modified octapeptide analogues of SST containing unnatural AA	(i) concentration-dependent antiproliferative effect after 24 h; (ii) the compound 4C (Orn^5^, Aib^6^) the most pronounced antiproliferative effects on HT-29		[109]
HT-29, SW480, LoVo and two cell lines to verify NE cell marker expression (DiFi and Colo320)	Exogenous SST and SST inhibitor cycloSST; ALDEFLUOR assay; FC; RT-PCR; crypt isolation	(i) after SST: Ʇproliferation but not ALDH+ population size or viability; (ii) co-cultured with SST1+ cells: Ʇ sphere-formation and Ʇcell proliferation of ALDH+ cells		[37]
HT-29 and other cell lines (MDA-MB-23, HepG2, HeLa, Lip-3)	A series of new analogues of BIM-23052, a linear SST analogue	(i) different concentration-dependent antiproliferative effect on cells after 24 h; (ii) all compounds bind well to SSTRs with preference to SST3 and SST5		[120]
HCT-116, HepG-2 and MCF-7	conjugated CTX-OCT loaded onto Ca-alginate-beads; DSC; FTIR; SEM; UV spectroscopy; cytotoxicity assay	CTX-OCT-Alg beads with gastro-resistant activity and efficiently deliver anti-cancer drugs to the higher pH environments of the colon with > antiproliferative activity vs. free drug		[136]
HCT-116, CT26 and others (Vero and BSC-40, HEK-293, B16, LO2, A549; U-2 OS, HeLa, SMMC-7721, SGC-790, MDA-MB-231 and MCF-7); BALB/c-nu mice, C57BL/6 and BALB/c mice with tumor cells	vaccinia VG9/(SST-14)_2_-HSA recombinant constructed by replacing SST fusion gene into TK locus of attenuated VG9 strain via homologous recombination	(i) a combined antitumor effect on SSTR+ cells; (ii) complete Ʇtumor in 3/10 mice after vaccinia VG9/TK^−^ or VG9/(SST-14)_2_-HSA, and ↑ survival of all mice in both groups; (iii) VG9/(SST-14)_2_-HSA is more effective in ↑ survival vs. VG9/TK; (iv) the oncolytic activity of vaccinia viruses not high enough in HCT-116; CT26 sensitive to all 3 viruses		[137]
SW620, HT-29, Colo205 and 17 other tumor cell lines; Colon 26 tumor fragments transplanted into BALB/C inbred mice	TT-232	(i) a 70% Ʇ tumor growth of Colon 26 tumor; (ii) ↑ apoptosis in HT-29 and SW620 (7× ↑ AI in HT-29 cells); (iii) Ʇ tyrosine kinase (75%) after 24 h in SW620, correlated well with ↓ proliferation and ↑ apoptosis; (iv) p53-independent apoptotic effect	↓ Cell proliferation Ʇ Tumor growth ↑ Cell apoptosis	[121]
transplantable murine colon 38 cancer	OCT (SMS, Sandostatin) and Mel; BrDU incorporation, weight of tumors; TUNEL; AI, LI	(i) after both SMS and Mel: ↓ LI and ↑ AI; (ii) AI in the group treated jointly with SMS and Mel < in groups treated separately with SMS or Mel; (iii) proliferation/apoptosis ratio < in the group treated with SMS or Mel		[151]
HT-29	^3^H-labeled heptapeptide somatostatin analogue TT-232	(i) (+) membranous and nuclear expression; (ii) low-affinity SSTRs in such cells, which might mediate the apoptosis-inducing effect		[133]
HT-29, HCT-15, HCT-116 and P388/R84; nude mice with tumor transplantation	AN-162, (DOX conjugated to SST carrier RC-121); RT-PCR	(i) (+) mRNA SST and high-affinity binding sites for SST in all cell lines; (ii) Ʇ HCT-116 and P388/R84 in S/G2 phase and ↑no. of apoptotic cells; (ii) ↓ volume of xenografts > its unconjugated components		[125]
WiDr with mp53	SMS 201-995 alone or in combination with 5-FU	(i) ↑ apoptosis, ↑ the % of cells with subdiploid DNA content; (ii) ↓ G0/G1 phase cells by 22.9% and 14.3% and G2/M by 14.3%; (iii) ↑ of 5-FU-induced S-phase Ʇ by a further 7.9%/12.9%/42.1% at 24/36/72 h, respectively		[135]
SW480	OCT; MTT and flow cytometric assays; microarray; WB	(i) Ʇ growth, ↑ apoptosis and arrested the G1 phase cells in a dose-dependent manner; (ii) ↑ 13 genes and ↓ 17 genes in Wnt/β–catenin pathway; (iii) ↑ pβ-catenin		[129]
Caco-2	SST; WB for Ku70, Ku86 and CLU; confocal microscopy; RT-PCR for Ku86	(i) ↑ Ku86 after 4 h; (ii) ↑ nCLU and ↑ Bax; (iii) ↑ binding between Ku70 and Ku86; (iv) ↑ the release of Bax from the Ku70/nCLU complex; (v) Ʇ proliferation after 24 h; (vi) restore apoptosis		[146]
SW480	OCT; apoptosis-DNA ladder assay; WB; RT-PCR; IHC	(i) ↑ SST2/SST5-induced apoptosis; (ii) ↑ accumulation of β-catenin in plasmalemma; (iii) Ʇ TCF-dependent transcription, and ↓ cyclin D1 and c-Myc; (iv) role in GSK-3β activation		[152]
Caco-2	OCT; FC and Sub-G1 fraction detection	(i) ↑ the proportion of apoptotic cells and ↓ cell proliferation; (ii) ↑ DNA fragmentation		[97]
A rat colonic ac implanted in female C57BL/6JBom-nu mice	OCT + galanin + serotonin	(i) ↓ the tumor volume, wet weight and relative volume density of BVs vs. C; (ii) ↑ AI in mice	↓ Cell proliferation ↑ Apoptosis ↓ Angiogenesis	[153]
human colon cancer cells injected in nude mice	OCT + galanin + serotonin vs. LV/5-FU	(i) ↓ the PI and the no. of tumor BVs; (ii) ↑ AI in the mice treated with both LV/FU-triple therapy and with triple therapy only vs. LV/FU-treated mice		[154]
SW620 cells implanted of the female nude mice (C57BL/6JBom-nu)	OCT + galanin + serotonin	(i) ↓ tumor volume, wet weight, PI and no. of tumor BVs in the treated animals; (ii) ↑ AI in the treated mice		[155]
human colon cancer cells injected in nude mice	OCT + galanin + serotonin; IHC, TUNEL; computed image analysis	(i) ↓ PI and the no. of tumor BVs in the mice; (ii) ↑ AI in the treated mice		[156]
human colon cancer cells injected into nude mice	OCT + galanin + serotonin vs. 5-FU/LV-irinotecan vs. 5-FU/LV-oxaliplatin	(i) no difference between the 3 groups regarding tumor proliferation, apoptosis, BVs density, EGF and VEGF expression		[157]
human colon cancer cells injected in nude mice; IHC; TUNEL; computed image analysis	OCT + galanin + serotonin vs. 5-FU/LV	(i) no difference between tumors treated with 5-FU/LV or triple therapy regarding the volume and weights of the tumors, apoptotis, proliferation, VEGF indices, BVs density; (ii) ↓ LI of EGF in the tumors treated with triple therapy vs. 5-FU/LV		[131]
Colo205 and HT-29	Four component peptides of DRF 7295 (VIP + bombesin + SP + SST)	(i) ↓ cAMP; (ii) ↓ EGF-dependent proliferation and the pMAPK (pERK1/2); (iii) ↑ p53 and ↓ Bcl-2 levels (in Colo205); (iv) Ʇ VEGF secretion and ↑ caspase-3 (in HT-29); (v) ↓ capillary tube like formation in ECs		[159]

Legend: (+)/(−): positive/negative expression/correlation; </>: lower/higher; ↑/↓: significant increased/decreased expression/upregulation/downregulation; Ʇ: arrest/blockade/inhibition/inactivation; A549: human lung ca cell line; AA: amino acids; ac: adenocarcinoma; AI: apoptotic index; ALDH: aldehyde dehydrogenase; Alg: alginate; B16: murine melanoma cell line; BrDU: 5-bromo-2′-deoxyuridine; BVs: blood vessels; C: control, normal colon mucosa; ca: carcinoma; Caco: 2-colorectal ca cells; C-26: human colon ac cells; C170: human colon ca cells; CEA: carcinoembryonic antigen; Colo205: colon ca cells; Colo320: human colorectal ac cells which secrete NE-like markers; COX: 2-cyclooxygenase-2; CRC: colorectal ca; CT26: murine colon ca; CTX: cetuximab; CX1: human colon ac cell line; cycloSST: cyclosomatostatin; DiFi: human colorectal ac cell line; DOX: doxorubicin; DSC: differential scanning calorimetry; EECs: enteroendocrine cells; EGF: epidermal growth factor; EIA: enzyme immunoassay; EM: immunoelectron microscopy; ENS: enteric nervous system; FC: flow cytometry; FTIR: Fourier-transform infrared spectra; 5-FU: 5-fluorouracil; GSK-3β: glycogen synthase kinase 3β; h: hour; ^[3H]^thymidine: tritiated thymidine; HCT-15, HCT-116: human colon ac cells; HEK-293: human embryonic kidney cells; HeLa: cervical cancer cells; HepG-2: human hepatocellular ca cells; HT-29: human colorectal cancer cells; IC: inhibitory concentration; IEC-6: non-transformed small intestinal cells from rat; IF: immunofluorescence; IFN: γ-interferon gamma; IHC: immunohistochemistry; IL-2: interleukin 2; LI: index of cell proliferation, labeling index; Lep-3: normal human diploid cell line; LIM 1215, LIM 1863, LIM 2405, LIM 2412, etc.,: human colon ac cells; LO2: human non-tumor hepatic cells; LoVo: human colon ca cells; LV: leukovorin; μM: micromole; MAPK: mitogen-activated protein kinase; MCF-7: human mammary ac cells; MDA-MB-23: human breast ac cells; Mel: melatonin; mp53: mutated p53; MTT-3-(4,5-imethyl thiazol-2-yl)-2, 5-diphenyl tetrazolium bromide, colorimetric assay of cellular respiration; nCLU: nuclear clusterin; NE: neuroendocrine; no.: number; NPs: neuropeptides; OCT: octreotide; p: phosphorylated; P388/R84-DOX: resistant mouse leukemia cells; PGE2: prostaglandin E2; PI: proliferating index; PTP: phosphotyrosine phosphatase; ref. no.—number of references; RT-PCR: real-time polymerase chain reaction/reverse transcription PCR; SEM: scanning electron microscopy; SGC-7901: human gastric ca cells; SMMC-7721: human hepatoca cells; SP: substance P; SST: somatostatin; SSTRs (SST1-5): somatostatin receptors 1–5; SW480, SW620: human colon ac cells; TCF: T cell factor; TRAP: telomeric repeat amplification protocol; TUNEL: terminal deoxynucleotidyl transferase biotin: dUTP nick and labeling; U-2 OS: human bone osteosarcoma cell line; VEGF: vascular endothelial growth factor; Vero and BSC-40: both African green monkey kidney epithelial cells; VIP: vasoactive intestinal peptide; WB: Western blot; WiDr: colon cancer cell line with mp53 which is derived from the same patient as HT-29 cells; wt: wild-type; X56: human colon ac cells.

**Table 2:** Potential antitumor activity of SST and SSAs in non-endocrine colorectal cancer (CRC) in vivo.

Antitumor Effect	Material (No. of Cases) and Methods (SST/SSA, Techniques)	Findings	Mechanism of Antitumor Activity/Clinical Significance	Ref. No.
↓ proliferation	Non-endocrine solid tumors (8); SMS 201-995	↓ in basal and arginine-stimulated sGH and sIGF-1	↓ sGH and ↓ sIGF-1 levels	[103]
	CRC (16); Sandostatin; a phase II study	(i) SD in 4 pts for 3–9 months; (ii) median survival—8 months; (iii) subjective improvement with a ↓ in pain	(i) ↑ Survival; (ii) disease stabilization	[104]
	RC (12); SMS 201-995; IHC	(i) ↓ Ki-67 tissue expression in 33% of pts; (ii) ↓ CEA in 50% of pts	↓ Ki-67 tissue expression	[101]
	CRC (25) and C (16); Sandostatin; IHC	↓ PCNA-MPI in 6/10 treated pts	↓ PCNA tissue expression	[102]
	CRC (24); LAN; ELISA	the highest doses seemed to maintain ↓ serum IGF-1; with the lowest doses, a “rebound” IGF-1 levels during therapy	No antitumor activity or tumor marker reduction	[105]
	CRC (75); OCT; ^[3H]^thymidine LI; FC; ELISA	(i) ↓ in the mean % of the S-phase fraction; (ii) ↓ sIGF-1; (iii) EGF and GH levels not affected	(i) Ʇ Cell cycle; (ii) ↓ sIGF-1	[106]
	CRC (12) and C (12); IHC; computer image analysis	(i) ↓ SST(+) cells and CSI in CRC vs. C; (ii) the nuclear volume of these cells did not differ vs. C	(i) ↓ Colonic content of SST in CRC vs. C; (ii) ↓ secretory activity (no antitumor activity)	[94]
	CRC (35) with LM (25); iEM; IHC	(i) ↑ SST in well-differentiated vs. poorly differentiated tumors	↓ SST correlated with poor grading and poor prognosis (no antitumor activity)	[95]
	CRC mirror biopsies (90); iEM	↓ SST tissue expression	↓ SST and ↑ or ectopic expression of other NPs may indicate the preneoplastic nature of the tissues	[96]
	CRC (79); IHC	(i) cyclin E LE > in low SST group vs. high and middle groups; (ii) CDK2 LE > in low SST group vs. high SST group; (iii) (+) correlation between the integral ratio of gastrin/SST and the cyclins (D1, E, A) and CDK2, CDK4 expression	(i) Abnormal tissue expression of cyclins and CDKs; (ii) the regulatory site of SST may be at the entrance of S phase	[107]
	CRC (34) and C (6/41) (children/adults) (TMA); CRC (13) and C (14/20) (IHC); CRC (12) and C (12/12) (qRT-PCR; IHC)	(i) ↓ SST mRNA in CRC vs. C (adults); (ii) ↑ ratio of SST(+) cells in children vs. CRC	*SST* gene promoter hypermethylation	[97]
	C (5), and matched CRC (5); qRT-PCR	(+) in all the C; (−) in matching CRC samples	Monitoring the rate of NCs maturation and SCs quiescence	[37]
↑ apoptosis	CRC (53) and tumor-neighboring mucosa with hyperplasia; IHC	(i) (+) SST in 84.6% CRC vs. 88.5% tumor-neighboring mucosa; (ii) SST coexpression with Bcl-2	Bcl-2 expression	[33]
	CRC (62); IHC	(i) LE of Bax in SST high and intermediate groups > low expression group; (ii) LE of Bcl-2 in SST high and intermediate expression groups < low expression group	Bax and Bcl-2 expression	[149]
	CRC (79); IHC; nested RT-PCR	(i) (+) correlation between SST mRNA and protein expression; (ii) AI in SST high and moderate expression groups > low expression groups; (iii) (+) LE of Fas, caspases 8/3 in SST high and moderate expression groups > low expression group	Fas, caspase 8 and caspase 3 expression	[150]
↓ angiogenesis	CRC (35); OCT; IHC; ELISA	(i) ↓ VEGF (*t*/*s* level); (ii) (+) correlation between t/s VEGF expression	↓ VEGF	[165]

Legend: (+)/(−): positive/negative expression/correlation; </>: lower/higher; ↑/↓: significant increased/decreased, reduced expression/serum level; Ʇ: inhibition, blockade; AI: apoptosis index; C: control, normal colon mucosa; ca: carcinoma; CC: colon carcinoma; CDK: cyclin-dependent kinase; CEA: carcinoembryonic antigen; CRC: colorectal carcinoma; CSI: cell secretory index; EGF: epidermal growth factor; ELISA: enzyme-linked immunosorbent assay; FC: flow cytometry; GH: growth hormone; iEM: immunoelectron microscopy with immunogold staining; IF: immunofluorescent microscope; IGF-1: insulin-like growth factor 1; IHC: immunohistochemistry; LAN: lanreotide; LE: level of expression; LI: labeling index; LM: liver metastasis; NCs: neuroendocrine cells; no.: number; NPs: neuropeptides; OCT: octreotide; PCNA-MPI: proliferating cell nuclear antigen–maximum proliferating index; pts: patients; RC: rectal carcinoma; s: serum level; SCs: stem cells; SD: stable disease; SSAs: somatostatin analogues; SST: somatostatin; t: tissue expression; TMA: tissue microarray; qRT-PCR: quantitative real: time PCR-VEGF: vascular endothelial growth factor; vs.: versus; ref. no.: number of references.

**Table 3:** The anti-inflammatory activity of SST and SSAs in various models of colitis.

Model of the Study	Material (No. of Cases) and Methods	Findings	Potential Role in IBD	Ref. No.
In vivo (Human)	Various GIT diseases (including UC), C; RIA	a postprandial ↑ SST in all pts and age-matched C, especially ↑↑ in pts with active UC (176 ± 17 pg/mL), IBS (194.4 ± 20.4 pg/mL) and duodenal ulcer (159 ± 20 pg/mL)	The variations in circulating IR SST concentrations may be of pathophysiologic importance	[194]
	Idiopathic IBD (UC, CD) and C; RIA	(i) SST-28 the major IR species; (ii) ↓ SST in the mucosa-submucosa of the descending colon in UC and in CD vs. C; (iii) SST levels: in severe < minimal colitis	Consistent with morphologic studies, which have suggested ↓ of EECs in UC	[191]
	CD (25); UC (25); CRC (25); IHC	(i) ↓ SST cells in CD and UC; (ii) the distal colon tended to contain >IR cells than the proximal colon did; (iii) ↓ (+) EECs in IBD vs. CRC; (iv) inverse correlation between (+) cells and the degree of inflammation in CD; (iv) ↓ (+) ganglion cells in CD	The decrease in SST-containing cells rather secondary to inflammation, however, may have some role in the pathogenesis of IBD	[192]
	UC, C; RIA	↑24 h amplitude, a ↑ average level and a longer peak level phase of plasma SST in UC vs. C	Potential defensive role of SST in IBD	[91]
	CD tissue (9); LAN; ELISA; ECs isolation and culture; FACS sorting; RT-PCR	after LAN: Ʇ IL-1β-stimulated 5-HT secretion in normal and Crohn’s-derived ECs	Inflammatory ECs are more sensitive to cytokine-mediated activation and have intact inhibitory mechanism via SSTR	[195]
	UC (5), CD (6), C; IHC for SST and other markers of innervation	↓ SST(+) nerve fibers surrounding submucosal arteries, from 22% to 1% (UC) and 2% (CD), but not perivascular mesenteric nerve fibers	Changes in the perivascular nerves may be responsible for the congestion, ulcerations and pain	[193]
In vivo (Animal)	male rats; acetic acid colitis; histology; RIA; OCT s.c. (10 μg/rat)	after OCT: (i) ↓ in mucosal damage; (ii) ↓ PAF, leukotriene B4 and VIP concentrations	Possible role of SST in the pathogenesis of colitis; the mechanism of OCT action—not determined	[199]
	male Wistar rats; TNBS colitis and C; OCT s.c. (2 × 10 μg × day/rat); IHC; WB; enzyme activities; culture ex vivo; ELISA	(i) max TNF-α produced at the 8th h, correlated with intense immunostaining of the external muscle layer; (ii) after OCT: ↓ TNF-α expression (staining and activity) and iNOS activity; (iii) ↓ submucosal MA TNF-α (+) and colonic production of IL-1β and IFN-γ	TNF-α regulation by OCT suggests that this drug might exert anti-inflammatory activities via smooth muscle cells	[198]
	old female C57BL/6 mice with *C. rodentium* (CR)- and DSS colitis; OCT; IHC; RIA	after OCT: (i) in CR colitis: ↑claudin-1 and claudin-3 expression vs. untreated CR infected mice; (ii) in DSS colitis: ↑claudin-3 expression vs. untreated DSS colitis mice	SST may play role in intestinal barrier protection by modulating TJ proteins expression	[202]
	male rats (30); TNBS colitis and C; IHC	(i) ↑ the densities of SST cells in TNBS vs. C group; (ii) ↑ the densities of mucosal leukocytes, B/T lymphocytes, T lymphocytes, B lymphocytes, MA/monocytes and mast cells in the TNBS group vs. C; (iii) (+) correlation between no. of specific immune cells and SST cells	Possible interactions between intestinal hormones (including SST) and immune cells	[197]
	male rats (40); TNBS colitis and C; IHC	↑SST cells in the TNBS group vs. control group	Potential effects of signaling substances produced during inflammation on hormone expression, resulting in abnormalities in EECs and SCs and their progenitors	[48]
	male rats (24); DSS colitis and C; IHC	(i) ↓ the densities of SST cells in DSS vs. C group; (ii) ↑ the densities of mucosal leukocytes, B/T lymphocytes, T lymphocytes, B lymphocytes, MA/monocytes and mast cells in the DSS group vs. C; (iii) (−) correlation between SST cells and no. of all types of immune cells	Possible interaction between intestinal hormones (including SST) and the immune cells	[200]
	wt C57BL/6 mice; DSS colitis; OCT; histology; EM; FITC: BT detection; IF	after OCT: (i) improvement of clinical symptoms and histopathology scores; (ii) ↓ epithelial barrier dysfunction and restores TJ complex; (iii) ↑ claudin-4 expression	The protective effect of SST is achieved by ↑ claudin-4 expression	[203]
In vitro	Caco-2 and HT-29; SST/OCT and cycloSST; ELISA; RNA extraction and RNA protection assay; MTT	after SST: (i) Ʇ the spontaneous and TNF-α-induced secretion of IL-8 and IL-1β mRNAs in dose-dependent manner, reaching >90% Ʇ at 3 nM; (ii) abrogation of the ↑ secretion of IL-8 and IL-1β after invasion by *Salmonella*; (iii) via SST2 and SST5 similar impact on the secretion of IL-8 and IL-1β; (iv) cycloSST completely Ʇ the SST- and OCT-induced ↓ effects; (v) no effect in cell viability	SST/SSAs are responsible for regulating the mucosal inflammatory response of intestinal epithelial cells following physiological and pathophysiological stimuli, including bacterial invasion	[196]
	Caco-2; CCK; FITC and PI; IF; TER; RT-PCR; SST-14 (1 nM)	after SST: (i) improvement of the barrier dysfunction and ↑ expression of occludin and ZO-1; (ii) ↓ the redistribution of TJ proteins due to LPS stimulation; (iii) ↓ the LPS-induced phosphorylation of ERK1/2; (iv) ↓ mRNA of SST5 increased by LPS	SST protects the Caco2 monolayer barrier against LPS-induced TJ breakdown by ↓the activation of the ERK/MAPK pathway and suppressing the activation of SST5	[201]
	Caco-2; EPEC and TNF-α; OCT (1 μM); WB	after OCT: (i) ↑ claudin-1 and ↑ claudin-3 expression in EPEC-infected cells vs. untreated cells; (ii) in cells exposed to TNF-α: ↑ claudin-3 expression vs. untreated cells	SST may play role in intestinal barrier protection by modulating TJ proteins expression	[202]
	Caco-2 pretreated with TNF-α; SST (1 μM); SSTR agonist; TER; RT-PCR; WB	after SST: (i) ↑ claudin-4 expression via SSTR5 in TNF-α intervened cells; (ii) ↓ the phosphorylation levels of p38 and ERK1/2 to the basal level vs. C	The protective effect of SST followed activation of SST5 and subsequent Ʇ of the ERK1/2 MAPK pathway	[203]

Legend: ↑/↓: increase/(stimulation)/decrease (inhibition); ↑↑: very intense increase; (+)(−): positive/negative expression; Ʇ: arrest/blockade/inhibition/inactivation; BT: bacterial translocation; C: control; Caco-2: colorectal ca cells; CCK: cell counting kit-8; CD: Crohn’s disease; *C. rodentium*: *Citrobacter rodentium*; cycloSST: cyclosomatostatin; DSS: dextran sodium sulphate; *E. coli*: *Escherichia coli*; ECs: enterochromaffin cells; EECs: enteroendocrine cells; ELISA: enzyme-linked immunosorbent assay; EM: immunoelectron microscopy; EPEC: enteropathogenic *E. coli*; ERK1/2: extracellular signal-regulated kinase 1/2; FITC: fluorescein isothyocyanate; h: hour; 5-HT: serotonin; HT-29: human colorectal cancer cells; IBD: inflammatory bowel disease; IBS: irritable bowel syndrome; IF: immunofluorescence; IFN-γ: interferon gamma; IHC: immunocytochemistry; IL-1β: interleukin 1β; iNOS: inducible nitric oxide synthase; IR: immunoreactive; LAN: lanreotide; LPS: lipopolysaccharide; MA: macrophages; MAPK: mitogen-activated protein kinase (originally called ERK); MTT-3(4,5-imethyl thiazol-2-yl)-2, 5: diphenyl tetrazolium bromide, colorimetric assay of cellular respiration; NPs: neuropeptides; OCT: octreotide; PAF: platelet-activating factor; PI: propidium iodide; RIA: radioimmunoassay; RT-PCR: real-time polymerase chain reaction/reverse transcription PCR; s.c.: subcutaneously; SSAs: SST analogues; SST: somatostatin; SSTRs (SST1-5): somatostatin receptors 1–5; pts: patients; ref. no.: number of reference; TER: transepithelial electrical resistance; TJ: tight junction; TNBS: trinitrobenzene sulphonic acid; TNF-α: tumor necrosis factor-alpha; UC: ulcerative colitis; WB: Western blotting; ZO-1, -2, -3: zonula occludens, 1/2/3 proteins.

## Abbreviations

ALDH	aldehyde dehydrogenase
AKT	serine/threonine-protein kinase or protein kinase B (PKB)
APC	adenomatous polyposis coli
CGRP	calcitonin gene-related peptide
CD	Crohn’s disease
c-Met	tyrosine-protein kinase Met or hepatocyte growth factor receptor
CRC	colorectal cancer
CSCs	cancer stem cells
DSS	dextran sodium sulfate
ECL	enterochromaffin-like
EECs	enteroendocrine cells
ENS	enteric nervous system
ERK1/2	extracellular signal-regulated kinase 1/2
GH	growth hormone
GPCR	G protein-coupled receptor
5-HT	5-hydroxytryptamine (serotonin)
HNPCC	hereditary non-polyposis CRC
IBD	inflammatory bowel disease
IC50	half-maximal inhibitory concentration
IHC	immunohistochemistry
IL-6/12	interleukin-6/12
IFN-γ	interferon γ
ISH	in situ hybridization
MANEC/MiNENs	mixed adenoendocrine carcinomas
MAP	mitogen-activated protein kinase (originally called ERK)
MIN/MSI	microsatellite instability
NCs	neuroendocrine cells
NEN	neuroendocrine neoplasm
NET	neuroendocrine tumor
OCT	octreotide
OS	overall survival
PTP	protein phosphotyrosine phosphatase
SCs	stem cells
SH2	Src homology 2
SHP1/2	Src homology 2 domain phosphatase 1/2
SRCC	signet-ring cell carcinoma
SRIF/SRIH/SST	somatotropin-release inhibitory factor/SRI hormone/somatostatin
SSAs	somatostatin analogues
SSTRs	somatostatin receptors
TNBS	trinitrobenzene sulfonic acid
UC	ulcerative colitis
VIP	vasoactive intestinal peptide
Wnt	gene wingless *+* integrated or int-1
